# Sintilimab plus bevacizumab followed by resection in intermediate-stage hepatocellular carcinoma: a phase Ib clinical trial with biomarker analysis

**DOI:** 10.1136/bmjonc-2024-000578

**Published:** 2024-12-16

**Authors:** Hui-Chuan Sun, Xiao-Dong Zhu, Zi-Yi Wang, Qiang Gao, Yuan Ji, Ying-Hong Shi, Xiao-Ying Wang, Shuang-Jian Qiu, Cheng Huang, Ying-Hao Shen, Jian Zhou, Jia Fan

**Affiliations:** 1 Department of Liver Surgery and Transplantation, Key Laboratory of Carcinogenesis and Cancer Invasion (Ministry of Education), Liver Cancer Institute, Zhongshan Hospital Fudan University, Shanghai, China; 2 Department of Pathology, Zhongshan Hospital Fudan University, Shanghai, China

**Keywords:** Immunotherapy, Liver cancer

## Abstract

**Objective:**

This phase Ib trial aimed to assess the safety and efficacy of sintilimab plus bevacizumab (sintilimab/bev), followed by resection in patients with potentially resectable intermediate-stage hepatocellular carcinoma (HCC) and explore the clinical implications of circulating tumour DNA (ctDNA) and T cell receptor (TCR) repertoire.

**Methods and analysis:**

Eligible patients with intermediate-stage HCC received sintilimab/bev treatment. Patients with partial response or stable disease for at least two consecutive evaluations and technically resectable received hepatectomy. Postoperatively patients continued to receive sintilimab/bev until tumour recurrence or intolerable toxicities for up to 12 months. The primary endpoints were treatment safety and event-free survival (EFS). Plasma ctDNA measurements and TCR repertoire were analysed.

**Results:**

30 patients were enrolled. 17 (56.7%) patients received liver resection. Grade 3 treatment-related adverse events occurred in seven patients (23.3%). No grade 4/5 AE or postoperative mortality was observed. The median EFS of the 30 patients was 16.3 months (95% CI 13.4 to 19.2). The 12-month and 24-month survival rates were 93.2% and 82.0%, respectively. Of the 17 patients who received hepatectomy, the median recurrence-free survival was 14.1 months (95% CI 8.9 to 19.4). A lower ctDNA measurement and higher TCR repertoire were associated with better tumour response or patients’ survival.

**Conclusions:**

The study suggested systemic therapy with sintilimab/bev was safe and effective in patients with intermediate-stage HCC, and resection in selected patients was associated with improved survival. ctDNA measurement and TCR repertoire may help identify patients who may benefit from sintilimab/bev treatment and patients with a higher risk of tumour recurrence.

**Trial registration number:**

NCT04843943.

**WHAT IS ALREADY KNOWN ON THIS TOPIC**

**WHAT THIS STUDY ADDS**

- The study suggested systemic therapy with sintilimab/bev followed by liver resection in selected patients was safe for intermediate-stage HCC. Selecting patients who respond to systemic therapy for surgical resection may improve event-free survival. Circulating tumour DNA (ctDNA) measurement and T cell receptor (TCR) repertoire may help identify patients who benefit from sintilimab/bev and patients with a higher risk of tumour recurrence.

**HOW THIS STUDY MIGHT AFFECT RESEARCH, PRACTICE OR POLICY**

- Conversion therapy with sintilimab/bev in potentially resectable intermediate-stage HCC patients was safe. The treatment approach of systemic therapy combined with active surgical treatment in patients with successful downstaging may provide greater survival benefits than systemic therapy alone. The present study also revealed that ctDNA measurement and TCR repertoire were promising in stratifying patients who may benefit from sintilimab/bev treatment and identifying patients with a higher risk of tumour recurrence.

## Introduction

 Primary liver cancer, mostly comprising hepatocellular carcinoma (HCC), is the seventh most common cancer and the third-leading cause of cancer death worldwide.^1^ 64% of HCC patients in China are initially diagnosed at the intermediate or advanced stage.^2 3^

Immune-checkpoint inhibitors-based combination therapies are currently the standard of care for advanced or unresectable HCC.^4^ The combination of sintilimab (an anti-PD-1 antibody) and IBI305 (a bevacizumab biosimilar), known as sintilimab/bev, was approved in China as a first-line systemic therapy for unresectable or metastatic HCC.^5^ Although several reports have demonstrated upfront resection for intermediate-stage, HCC provided a better survival compared with transarterial chemoembolisation (TACE),^6^ resection for intermediate-stage HCC remains controversial. Recently, several retrospective studies suggested that systemic therapy provided an opportunity for curative treatment, mostly resection, for patients with intermediate/advanced stage or initially unresectable HCC and may lead to long-term disease-free survival.^7^,^10^ In this regard, we conducted a prospective study to investigate the safety and efficacy of systemic therapy using sintilimab/bev in patients with intermediate-stage HCC, followed by resection in patients who have benefited from sintilimab/bev treatment. Meanwhile, blood samples were collected to explore the clinical implications of circulating tumour DNA (ctDNA) and T cell receptor (TCR) repertoire. To our knowledge, this is the first study to demonstrate the efficacy of an approved regimen of bevacizumab-based combination therapy, followed by resection in patients with intermediate-stage HCC.

## Methods

### Study design and participants

This was an open-label, single-centre, single-arm, phase Ib clinical trial (NCT04843943) online supplemental file 1.

Treatment-naïve patients with potentially resectable intermediate-stage (Barcelona Clinic Liver Cancer stage B or Chinese Liver Cancer stage IIa and IIb) HCC were enrolled. Other eligibility criteria are listed in online supplemental material 1. The criteria of ‘potential resectable’ HCC were assessed by the investigators and the primary criteria are non-diffuse and non-infiltrative tumours, and tumour burden did not exceed 70% of liver volume. Oesophagogastroduodenoscopy examination is mandatory before enrolment. Patients with severe and untreated oesophageal or gastric varices are not eligible. Signed informed consent was obtained from each patient.

### Procedures

Enrolled patients received sintilimab (200 mg, intravenous, day 1) and bevacizumab (15 mg/kg, intravenous, day 1) every 3 weeks until intolerable toxicities or disease progression. Tumour response and resectability were evaluated by contrast-enhanced MRI or CT at baseline and every 6 weeks per Response Evaluation Criteria in Solid Tumors (RECIST) V.1.1 and modified RECIST in the initial 6 months and every 9 weeks thereafter. Patients assessed as partial response (PR) or stable disease (SD) per RECIST V.1.1 criteria for at least two consecutive evaluations and technically feasible for liver resection were recommended for hepatectomy. In 4–8 weeks after hepatectomy, patients continued to receive adjuvant sintilimab/bev therapy until tumour recurrence or intolerable toxicities for up to 12 months. In case of complete response (CR) based on RECIST V.1.1 or being technically infeasible for resection, sintilimab/bev treatment continued until intolerable toxicities or tumour progression, for up to 24 months.

### Hepatectomy

Patients will be assessed by a multidisciplinary team. If resection is recommended, the patient will receive sintilimab monotherapy for one cycle, therefore, at the time of hepatectomy sintilimab was discontinued for more than 3 weeks, and bevacizumab was discontinued for more than 6 weeks. Hepatectomy and postoperative management were carried out as previously reported.^11 12^ Surgical resection combined with concurrent intraoperative ablation therapy is allowed. Surgical specimens were collected for further analysis.

### Patient outcomes

Primary endpoints were treatment safety and event-free survival (EFS) (time from enrolment to tumour progression, tumour recurrence after hepatectomy or death from any cause). Treatment safety evaluation included adverse events (AEs), treatment-related AEs (TRAEs) and serious AEs (SAEs), which were defined and graded until 30 days after the last dose per Common Terminology Criteria for Adverse Events (CTCAE) V.5.0. Operation safety was evaluated by intraoperative blood loss and postoperative events, which included posthepatectomy liver failure (PHLF) grades based on the International Study Group of Liver Surgery (ISGLS) criteria^13^ and postoperative complications based on the modified Clavien-Dindo system.^14^ Secondary endpoints included R0 resection rate, pathologic response, objective response rate (ORR), recurrence-free survival (RFS) in patients underwent hepatectomy, progression-free survival (PFS) in patients who did not receive resection and overall survival (OS) (time from enrollment to death from any cause). EFS, ORR, RFS and PFS were assessed by investigators per RECIST V.1.1. ORR was also evaluated per modified RECIST criteria. Exploratory endpoints were ctDNA analysis, TCR repertoire and pathological evaluations.

### Patient and public involvement

Patients or the public were not involved in the design, or conduct, or reporting, or dissemination plans of our research.

### Exploratory biomarker study

The protocol of blood sample collection, cell-free DNA (cfDNA) and genomic DNA isolation, ctDNA low-pass whole genome sequencing, targeted sequencing and bioinformatic analyses, and TCR repertoire analyses are shown in online supplemental material.

### Statistical analysis

In the ORIENT-32 study,^5^ the median PFS in the sintilimab/bev group was 4.6 months (95% CI 4.1 to 5.7). Assuming that surgery after sintilimab/bev treatment in selected patients would double the PFS with a median EFS of 9.2 months, a sample size of 29 patients was required to provide a power of 80% with a one-sided alpha of 0.025, considering 1 year of enrolment and 1 year of follow-up and allowing for a dropout rate of 20%. The sample size in this phase Ib study was fixed at 30.

The Simon’s two-stage design was applied. The assumption of the Simon’s two-stage design is that resection rates ≥30% and <10% are considered to be clinically effective and ineffective, respectively. The null hypothesis that the conversion resection rate is 30% was tested against a one-side alternative. Based on these assumptions, 12 patients were enrolled in the first stage. In case of a number of resection cases ≤1, patient enrolment would be terminated. In case >1 patient received resection in the first stage, the second stage was continued until 30 patients were enrolled. All statistical analyses used SPSS V.26.0 (IBM).

### Role of the funding source

The funder participated in the study design but had no role in data collection, data analysis, data interpretation or the writing of the report.

## Results

### Clinical outcome after treatment with sintilimab/bev combination

**Figure 1:**
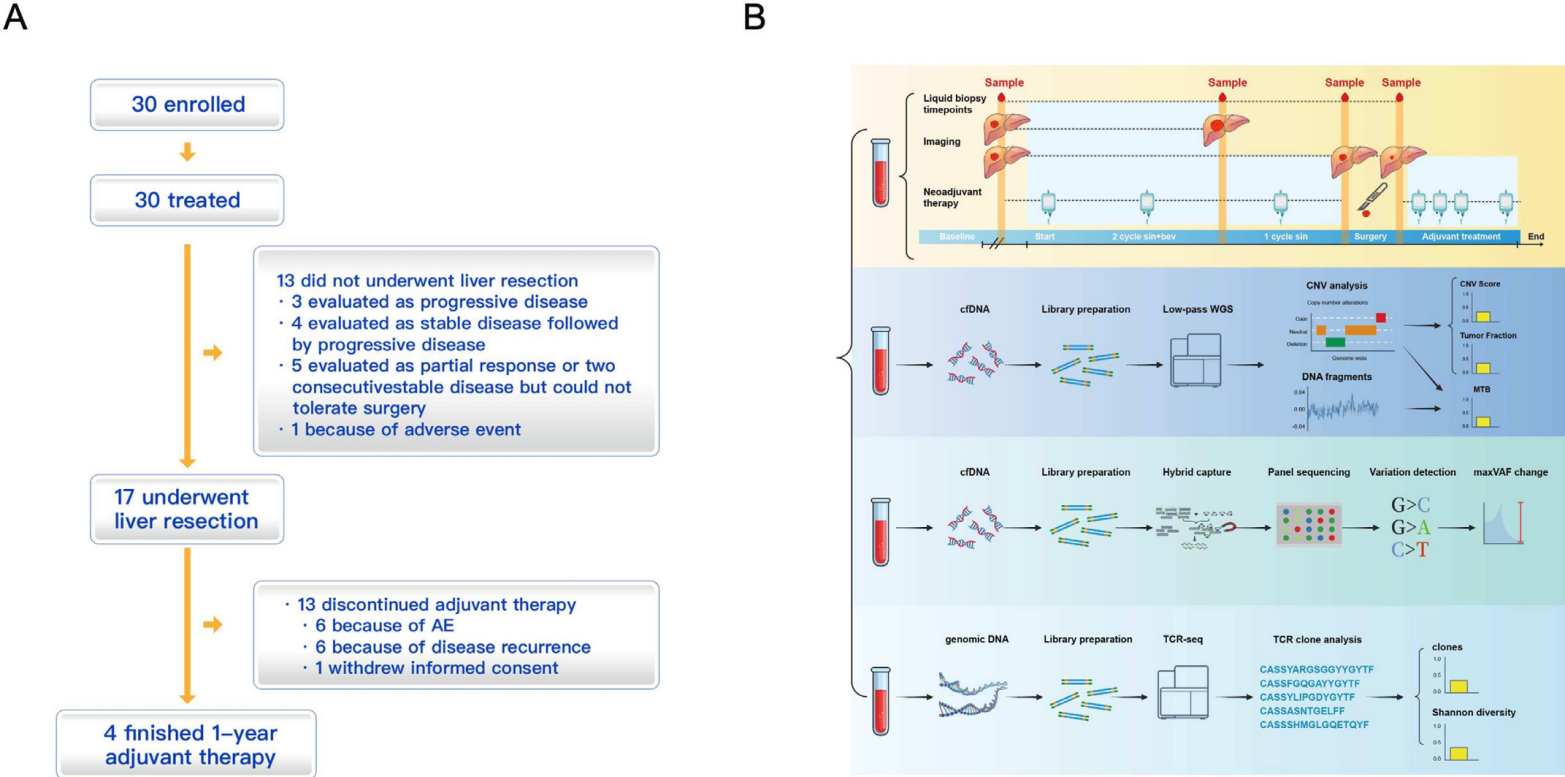
Trial design. (**A**) The flow diagram of the trail. (**B**) Patient enrolment, treatment and serial liquid biopsy for molecular response assessment. AE, adverse event.

Between 23 March 2021 and 19 January 2022, 30 patients were enrolled (figure 1A). (24/30) 80.0% had hepatitis B virus infection, the mean tumour size was 6.7 cm. (24/30) 80% of patients were beyond up-to-7 criteria or BCLC B2 stage according to Kinki criteria^15 16^ (table 1). No patient was classified as BCLC stage B3 because all the patients had Child-Push class A liver function.

**Table 1:** Demographic data of the patients in this study

	All subjects (n=30)
Sex (male/female), n	29/1
Mean age, years (SD)	59.1 (9.5)
ECOG performance status (0/1/2), n	24/6/0
Aetiology (HBV/HCV/non-viral), n	24/1/5
Tumour size, cm (SD)	6.7 (4.0)
Tumour number (2–3/≥4)	16/14
Kinki criteria, up to 7 (in/out), n	6/24
China liver cancer stage (IIa/IIb), n	16/14
Child-Pugh class (A/B), n	30/0
Baseline AFP≥400 ng/mL, n (%)	5 (16.7%)
Baseline PIVKA-II≥400 mAU/mL, n (%)	19 (63.3%)

AFP, alpha-fetoprotein; ECOG, Eastern Cooperative Oncology Group; HBV, Hepatitis B; HCV, Hepatitis C; PIVKA-II, Protein Induced by Vitamin K Absence-II.

**Figure 2:**
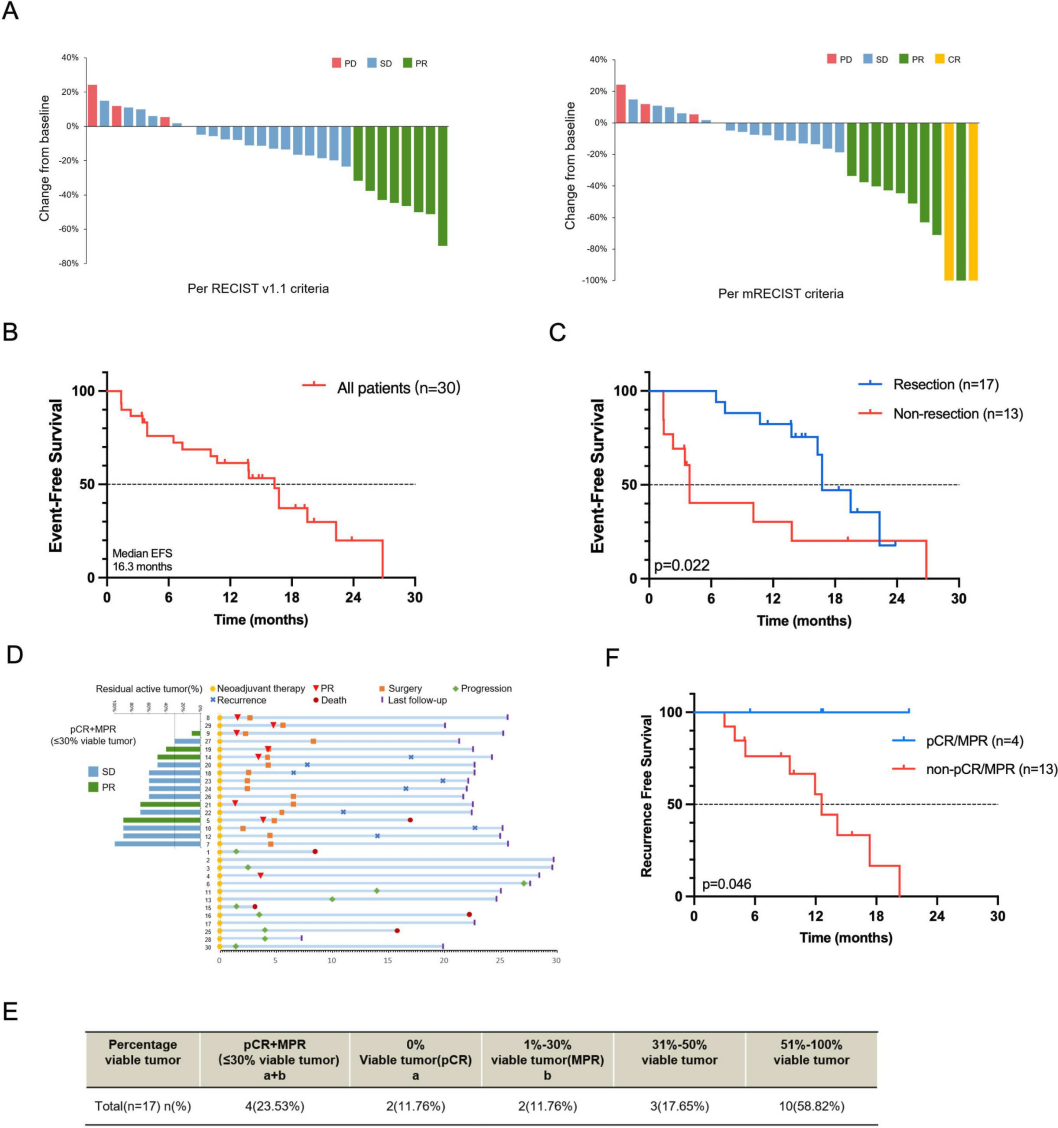
Summary of patients’ outcome. (**A**) Radiographic responses in all enrolled patients. (**B**) Event-free survival of all enrolled patients. (**C**) Event-free survival for patients who underwent liver resection or did not undergo liver resection (**D**) Swimmer plots of survival outcome in all enrolled patients and waterfall plots of pathological tumour regression (% viable tumour) in resected patients after conversion therapy. (**E**) Pathological responses in all resected patients. (**F**) Recurrence-free survival for patients who underwent liver resection with or without pathological complete response (pCR)/major pathological response (MPR). PD, progressive disease; PR, partial response; SD, stable disease.

At the data cut-off on 6 September 2023, the median observation time was 22.3 (IQR 20.9–24.8) months. All patients underwent at least one post-treatment imaging assessment. ORRs evaluated by RECIST V.1.1 and mRECIST before hepatectomy were 26.7% (8/30) and 36.7% (11/30) (online supplemental table 1 and figure 2A).

### Surgical resection

17 patients (56.7%, 7 PR and 10 SD per RECIST V.1.1) received R0 resection, rejecting the null hypothesis of a resection rate of less than 10%. No patient had delayed hepatectomy due to adverse effects related to sintilimab/bev treatment. The median time from the initiation of sintilimab/bev therapy to hepatectomy was 4.3 (IQR 2.3–5.6) months. Of them, 12 patients were BCLC stage B2 (beyond up to 7) at baseline, and three were downstaged from BCLC B2 to B1 before surgery.

All surgeries were open procedures, with 3 of them being major liver resections (≥3 segments). A total of 61 lesions were resected (n=48) or concurrently ablated (n=13). The median number of lesions for each patient was 3 (range 1–8). The mean tumour size of the largest tumour lesions resected was 4.4 (SD, 2.6) cm.

No postoperative death occurred. Median intraoperative blood loss was 100 (range 50–500) mL. The incidence of PHLF was 47.1% (8/17) per ISGLS criteria, and all were grade A. Six patients experienced postoperative complications per modified Clavien-Dindo criteria, including one grade I (postoperative ascites leading to delayed drainage tube removal), three grade II (infections requiring antibiotics) and two-grade IIIa (pleural effusion requiring additional puncture) complications. The median hospital stay after surgery was 10 (range 5–18) days.

### Follow-up

The median EFS of the 30 patients was 16.3 (95% CI 13.4 to 19.2) months (figure 2B), exceeding the expected median EFS of 9.2 months. 10 of the 13 (76.9%) patients who did not receive hepatectomy had progressive disease with a median PFS of 3.9 (95% CI 3.3 to 4.6) months. Eight of the 17 (47.1%) patients who received hepatectomy had tumour recurrence. The hepatectomy group had better EFS (p=0.022, figure 2C).

Five patients died. Of them, four did not receive hepatectomy (two because of tumour progression and two because of liver failure), and one case who received hepatectomy died from complications of a hip replacement surgery 11.9 months after hepatectomy (figure 2D). The median OS was not mature. The 12-month and 24- month survival rates of the 30 patients were 93.2% and 82.0%, respectively.

### Treatment safety

TRAE of any grade and grade 3 TRAEs occurred in 25 (83.3%) and 7 (23.3%) patients, respectively (online supplemental table 2). No grade 4/5 AE was observed. SAE occurred in nine (30%) patients. Of these, three were treatment related, including proteinuria (n=1, bevacizumab related), rash and hypoadrenocorticism (n=1 each, sintilimab related); six of them were probably treatment unrelated, including acute myocardial infarction (n=1), hypoproteinaemia (n=1), haemorrhage with tumour rupture (n=1), obstructive jaundice (n=2) and kidney dysfunction (n=1).

### Outcomes in patients received hepatectomy

Of the 17 patients who received hepatectomy, 2 patients (6.7%) had a pathological CR (pCR) and 2 (6.7%) had a major pathological response (MPR, residual viable tumour area/total tumour bed area ≤30%) (figure 2E). The median RFS was 14.1 (95% CI 8.9 to 19.4) months. Patients with a pathological response (pCR or MPR) presented with a better RFS compared those without a pathological response (p=0.046) (figure 2F). None of the 4 patients with a pathological response had tumour recurrence, while 8 out of the 13 patients without a pathological response had tumour recurrence (figure 2D). 11 patients discontinued sintilimab/bev treatment because of completion of 12 months of adjuvant treatment (n=4), or intolerable AE (n=6) or patient willing (n=1). Eight out of the 11 patients remained tumour-free at the time of data cut-off. So, 8 (26.7%) patients achieved drug-free and cancer-free survival for a median of 7.8 (range 3.0–20.0) months.

#### cfDNA mutation measurements and treatment efficacy

**Figure 3:**
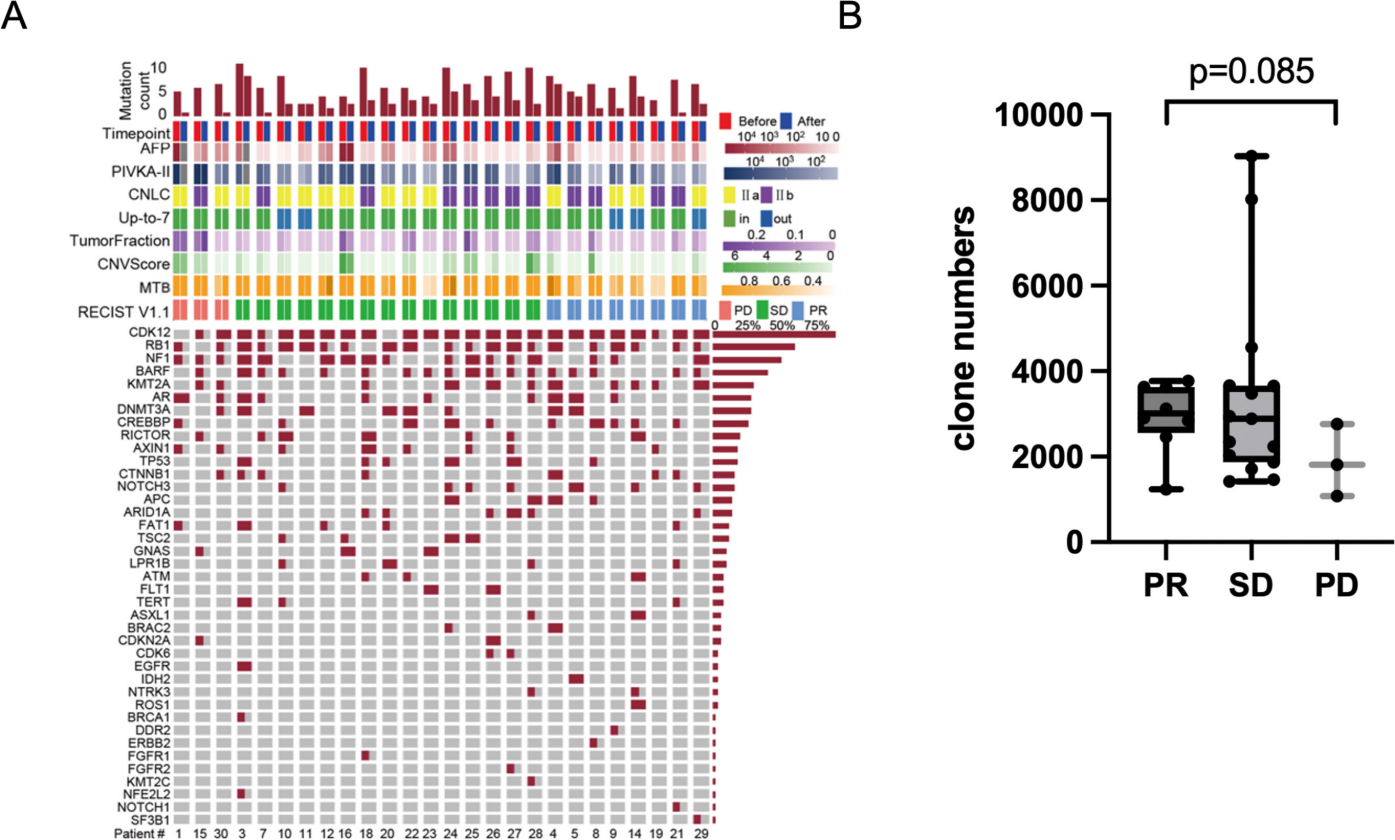
Overview of plasma ctDNA profiling and T cell repertoire by next generation sequencing (NGS). (**A**) Overview of plasma variants detected by comprehensive genomic profiling (CGP). (**B**) The difference of clone numbers between PR and PD patients. ctDNA, circulating tumour DNA; PD, progressive disease; PR, partial response.

26 and 14 blood samples were collected at baseline (1–3 days before sintilimab/bev treatment initiation), at postsystemic therapy (1–3 days after disease progression or 1–3 days before hepatectomy), and at postsurgery (4–6 weeks after surgery), resepectively (figure 1B). Blood samples were not collected from subjects #2. #6, #13 and #17, who were not evaluated as PD and did not undergo hepatectomy. EpiCGP 500 targeted sequencing was performed to detect mutations on cfDNA. The most frequently mutated genes were CDK12, RB1, NF1, BRAF and KMT2A, with mutation frequency of 0.87, 0.58, 0.48, 0.38 and 0.29, respectively (figure 3A).

**Figure 4:**
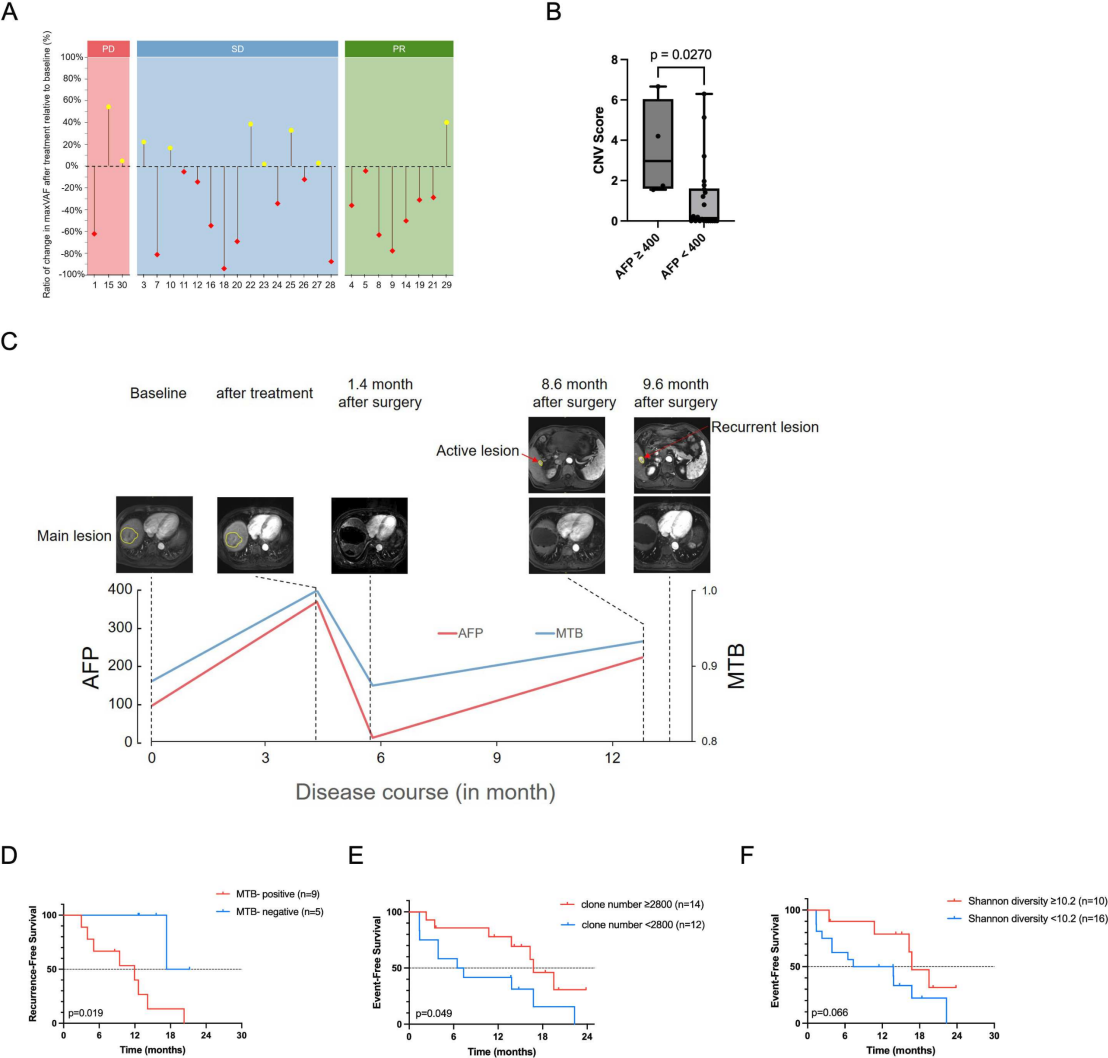
Predictive role of patient ctDNA features in tumour response and patients’ outcome. (**A**) Dynamic changes of ctDNA maxVAF from the baseline to postsystemic treatment in patients with different tumour response based on RECIST V.1.1. PR group showed the highest proportion of maxVAF decrease. (**B**) The difference of CNV score in patient with different AFP status. (**C**) Change of disease burden response to treatment based on MTB and MRI scan in subject #12. (**D**) Kaplan-Meier survival analysis shows the probability of RFS stratified by MTB status of postsurgery samples. (**E**) Kaplan-Meier survival analysis shows the probability of EFS stratified by TCR clone number of baseline. (**F**) Kaplan-Meier survival analysis shows the probability of EFS stratified by TCR diversity status of baseline. AFP, alpha-fetoprotein; ctDNA, circulating tumour DNA; CNV, copy number variation; EFS, event-free survival; MTB, molecular tumour burden; PD, progressive disease; PR, partial response; SD, stable disease; TCR, T cell receptor; VAF, variant allelic frequency; RECIST, Response Evaluation Criteria in Solid Tumors.

The maximum somatic variant allelic frequency (maxVAF), defined as the highest VAF among all ctDNA mutations identified in each cfDNA sample at both baseline and postsystemic therapy, was not associated with EFS; however, a decreased maxVAF from the baseline to postsystemic treatment was found in 7 out of 8 patients (88%) in the PR group, 9 out of 15 (60%) in the SD group and only 1 out of 3 patients (33%) in PD group (figure 4A).

#### cfDNA fragmentomes and treatment efficacy

Copy number variation (CNV) score, which was developed by the Shannon’s entropy formula and represents the overall fluctuation or disorder of the CNV on a genome-wide scale, at baseline, was higher in patients with higher alpha-fetoprotein level (≥400 ng/mL) (2.97 vs 0.14, p=0.027) (figure 4B). Furthermore, patients with CNV score ≥1.5 in baseline samples (n=10) or postsystemic therapy samples (n=6) had a poorer EFS compared with the remaining cases (median EFS: 3.9 vs 16.7 months, p=0.043; median EFS: 3.7 vs 16.7 months, p=0.001) (online supplemental figure 1A,B). Patients with objective response (n=8) had significantly lower CNV scores than those without objective response in postsystemic therapy samples (0.02 vs 1.24, p=0.041) (online supplemental figure 1C).

The tumour fractions indicating the amount of tumour-derived DNA fragments in plasma cfDNA were obtained from the analysis results of IchorCNA software. Elevated tumour fraction (TF, ≥10%) in either baseline (n=5) or postsystemic therapy samples (n=3) was associated with poorer EFS (median EFS: 3.5 vs 16.7 months, p=0.014; median EFS: 1.4 vs 16.7 months, p=0.003) (online supplemental figure 2A,B), and TF reduction between baseline and postsystemic therapy (TF decrease <0, n=5; TF decrease ≥0, n=21) was associated with better EFS (median EFS: 3.9 vs 16.7 months, p=0.035) (online supplemental figure 2C). Patients who achieved objective response (n=8) had lower TF in postsystemic therapy samples compared with those without objective response (0.00 vs 0.05, p=0.049) (online supplemental figure 2D).

The molecular tumour burden (MTB) is a probability value predicted by a machine learning model based on tumour fraction, CNV score and the ctDNA fragmentation distribution. Change of MTB after systemic treatment in patient #12 was presented in figure 4C and suggested that MTB was associated with tumour burden.

Patients with MTB-positive (≥0.5, n=9) in postsurgery samples were associated with poorer RFS (median RFS: 11.9 vs 19.3 months, p=0.019) (figure 4D). Besides, patients with either high CNV score (≥1.5, n=1) or high TF (≥5%, n=2) in the postsurgery samples tended to have shorter RFS than those with low CNV score or low TF (median RFS: 3.0 vs 14.1 months, p=0.001; median RFS: 7.8 vs 17.3 months, p=0.094, respectively) (online supplemental figure 3A,B).

### TCR repertoire

Patients with higher TCR clone numbers at baseline tended to have better tumour response per RECIST V.1.1 (PR vs PD, t-test, mean value: 2950 vs 1885, p=0.085) (figure 3B).

Patients with high TCR clone number (≥2800, n=14) and high Shannon entropy metric (≥10.2, n=10) at baseline had longer EFS than the others (median EFS: 16.7 vs 6.9 months, p=0.049; median EFS: 16.7 vs 10.6 months, p=0.066, respectively) (figure 4E,F). Patients with high clone number (≥2800) in postsystemic therapy samples had better EFS than the others (median EFS: 16.7 vs 3.7 months, p=0.005) (online supplemental figure 4A). These results indicated that TCR repertoire was associated with treatment efficacy and survival outcome.

## Discussion

This study suggested that sintilimab/bev treatment followed by hepatectomy in selected patients was safe and effective in intermediate-stage HCC (80% were beyond up-to-7 criteria). The treatment approach of systemic therapy combined with active surgical treatment in patients with successful downstaging may provide greater survival benefits than systemic therapy alone. This may be because the former treatment modality reduces the chance of tumour progression after treatment resistance develops and also provides some patients who respond to systemic therapy with the opportunity for curative treatment. The present study also demonstrated plasma ctDNA and TCR repertoire correlated to tumour response and patients’ survival.

Although TACE is the standard of care for intermediate-stage HCC, the present study was to explore a novel treatment sequence, that is, surgical resection after systemic therapy in the selected patients. The results demonstrated that this approach yielded a median EFS of 16.3 months (95% CI 13.4 to 19.2) in the whole cohort, which may be numerically better than 12.6 months in BCLC-B patients in the IMbrave150 study^17^ or 10.8 months in atezolizumab plus bevacizumab treated BCLC-B stage HCC in a real-world study,^18^ as well as those of TACE-treated (5.4 months) and hepatic artery infusion chemotherapy-treated (9.6 months) intermediate-stage HCC patients,^19^ or TACE in combination with systemic therapy (15.0 months) in the newly published EMERALD-1 study.^20^ Thus, the results support that this novel approach may provide a better outcome for intermediate-stage HCC patients compared with the existing treatment approaches.

The present study showed hepatectomy may provide an opportunity for curative outcome in the selected intermediate-stage patients after receiving systemic therapy, 10/30 (33.3%) remained tumour-free at the data cut-off date, and 8 (26.7%) patients achieved cancer-free and drug-free survival for 7.8 months. Kudo *et al* reported that 25 (22.7%) out of 110 patients with intermediate-stage HCC achieved clinical CR and drug-free survival after treatment with combination therapy of atezolizumab and bevacizumab followed by resection, RFA or TACE.^21^ Together with Kudo’s study, the present studies suggested that the BCLC stage B patients may achieve drug-free curative outcome after the novel approach.

Prediction of tumour response in HCC is difficult because current systemic therapies were not designed to target tumours with specific gene mutations. Our exploratory analysis showed a lower ctDNA measurement (maxVAF, CNV score, tumour fraction and MTB) associated with better tumour response or survival, which is consistent with our findings that mutations and fragmentomes detected after sintilimab/bev treatment associates with tumour response or EFS (figure 4). On the other hand, the present study did demonstrate TCR repertoire in the baseline or postsystemic therapy samples associated with tumour response and patients’ survival, which is consistent with previous reports.^22^,^26^ These findings support further investigation on ctDNA measurement or TCR repertoire in prediction or monitoring tumour response.

Molecular residual disease (MRD) is potentially able to guide adjuvant therapy after resection.^27 28^ The findings from our study showed patients with a higher MTB or CNV score or tumour fraction bearing higher risk of tumour recurrence. This approach does not require a high sequencing depth as the tumour-informed assay, but the sensitivity is not inferior to it.^29^,^31^ Tumour informed approach is a more popular method to detect MRD.^27 28 32^ However, tumour biopsy was not mandatory in this study and in clinical practice for HCC to avoid potential risk of bleeding or tumour seeding.^33^

This is a single-centre phase Ib study, and the majority of enrolled patients are HBV-related HCC, these characteristics limit the generalisability of the findings. This study had other limitations. First, a deeper tumour response could be achieved if continuous sintilimab/bev treatment is given while doctors were inclined to remove the tumour earlier to avoid tumour progression. Second, the value of resection in patients who responded to systemic treatment needs to be further evaluated in a controlled study (NCT04649489). Third, biomarkers in predicting and monitoring tumour response or MRD are not extensively compared with the existing criteria due to the limited sample size.

In summary, the results demonstrated efficacy of systemic therapy with sintilimab/bev treatment in patients with intermediate-stage HCC, followed by resection in those who met predesignated criteria. In addition, ctDNA measurement and TCR repertoire are promising in stratifying patients who may benefit from sintilimab/bev treatment and identifying patients with a higher risk of tumour recurrence.

## Supplementary material

10.1136/bmjonc-2024-000578 online supplemental file 1

10.1136/bmjonc-2024-000578 online supplemental file 2

## Data Availability

Data are available on reasonable request.
