# Supplementary material for: Sintilimab plus bevacizumab followed by resection in intermediate-stage hepatocellular carcinoma: a phase Ib clinical trial with biomarker analysis
Source: BMJ Oncol. 2024 Dec 16;3(1):e000578. doi: 10.1136/bmjonc-2024-000578 (PMC11880783; doi:10.1136/bmjonc-2024-000578)
Supplement: online supplemental file 1 [file bmjonc-3-1-s001.docx]

**CLINICAL STUDY PROTOCOL**

**STUDY TITLE:** Sintilimab combined with IBI310 (bevacizumab biosimilar) as a conversion therapy in potentially resectable intermediate-stage hepatocellular carcinoma: a phase Ib trial.

# Protocol Synopsis

| **Sponsor** | Zhongshan Hospital, Fudan University |
| --- | --- |
| **Test drugs** | Sintilimab: A recombinant fully human anti-programmed death receptor-1 (PD-1) monoclonal antibody.  IBI310 (Bevacizumab biosimilar): A recombinant anti-VEGF humanized monoclonal antibody. |
| **Protocol title** | Sintilimab combined with IBI310 (bevacizumab biosimilar) as a conversion therapy in potentially resectable intermediate-stage hepatocellular carcinoma: a phase Ib trial |
| **Developmental**  **phase** | Phase Ib |
| **Study objectives** | **Primary Objectives:**   - To evaluate the safety and efficacy of sintilimab/bev followed by resection in patients with intermediate-stage HCC. - To assess Event-Free Survival (EFS) as evaluated by RECIST 1.1.   **Secondary Objectives:**   - To determine the conversion to resectability rate (R0 resection rate). - To assess the pathological response rate. - To assess the Objective Response Rate (ORR) as evaluated by RECIST 1.1. - To assess Recurrence-Free Survival (RFS) in participants who receive surgical resection, as per RECIST 1.1. - To assess Progression-Free Survival (PFS) in participants who do not receive surgical resection, as per RECIST 1.1. - To assess overall survival (OS) in participants who receive surgical resection. - To assess OS in participants who do not receive surgical resection. - To assess OS in all participants.   **Exploratory Objective:**   - To investigate the value of blood biomarkers, including circulating tumor DNA (ctDNA) and T cell receptor (TCR) repertoire in predicting treatment efficacy and adverse reactions. |
| **Study design** | This is a single-arm phase Ib study focusing on patients with untreated potentially resectable intermediate-stage HCC (CNLC-IIa and IIb). It aims to evaluate the safety and efficacy of Sintilimab in combination with a IBI310 (Bevacizumab biosimilar) as the conversion therapy in these patients. The primary endpoints are safety (including the safety of drug treatment and surgical resection) and event-free survival (EFS), with secondary endpoints including R0 resection rate, pathological remission rate, ORR.  For the first 6 months after starting medication, effectiveness and surgical resection evaluations are performed every 6 weeks based on RECIST1.1 criteria, then every 9 weeks thereafter:  1) For CNLC-IIa stage patients with technically resectable tumors, after two cycles of medication leading to Partial Response (PR) or Stable Disease (SD) and deemed surgically resectable, stop Bevacizumab biosimilar treatment, continue with one cycle of Sintilimab (ensuring at least 6 weeks between the last dose of Bevacizumab biosimilar and surgery), followed by pre-surgery evaluation, surgical resection, and post-surgery 4-8 weeks of Sintilimab combined with Bevacizumab biosimilar adjuvant therapy until recurrence, intolerable toxicity, or a maximum of 12 months (16 cycles).  For CNLC-IIb stage patients or those with technically unresectable tumors that after medication show PR or two consecutive SD evaluations and are deemed surgically resectable, follow the same procedure.  2) If evaluated as Progressive Disease (PD) during treatment, it is recommended to exit the study and choose another treatment option.  3) If surgical evaluation deems the tumor unresectable or if evaluated as Complete Response (CR), continue the original treatment plan until toxicity becomes intolerable, disease progression, or death, up to a maximum of 24 months (32 cycles).  Based on the Simon two-stage design, this study predicts a conversion resection rate greater than 30%, considering the conversion treatment ineffective if below 10%. Initially, 12 participants are enrolled; if fewer than one conversion resection occurs, enrollment halts. Should more than one conversion resection be observed, the second stage proceeds until 30 participants are enrolled. The treatment is deemed ineffective if fewer than five total conversion resections are observed across both stages.  The study will also explore the predictive biomarkers of treatment efficacy. Peripheral blood samples of 10 mL will be collected from participants within 14 days prior to their first medication administration and again before surgery or upon disease progression. |
| **Inclusion criteria** | Participants eligible for this study must meet all the following criteria:  1. Able to provide informed consent and willing to sign an approved consent form before any trial-related procedures are conducted.  2. Male or female, aged ≥18 and ≤75 years.  3. Hepatocellular carcinoma diagnosed with histology/cytology or clinically according to Guidelines for the Diagnosis and Treatment of Hepatocellular Carcinoma (2019 Edition).  4. Intermediate-stage HCC (BCLC stage B or CNLC stage IIa/IIb) deemed potentially resectable upon the investigators’ assessment.  5. No prior treatment for HCC.  6. Child-Pugh class A.  7. Eastern Cooperative Oncology Group Performance Status (ECOG PS) score of 0-1.  8. Expected survival time of more than 6 months.  9. At least 1 measurable lesion according to RECIST 1.1 criteria.  10. Adequate bone marrow and organ function, including:  1) Absolute Neutrophil Count (ANC) ≥1.0×10^9^/L without the use of granulocyte colony-stimulating factor within the last 14 days;  2) Platelets ≥75×10^9^/L without transfusion within the last 14 days;  3) Hemoglobin ≥9 g/dL without transfusion or the use of erythropoiesis-stimulating agents within the last 14 days;  4) Total bilirubin ≤2.0 times the upper limit of normal (ULN);  5) Albumin ≥2.8 g/dL;  6) Aspartate aminotransferase (AST) and Alanine aminotransferase (ALT) within ≤5 times ULN;  7) Creatinine ≤1.5 times ULN and creatinine clearance (calculated using the Cockcroft-Gault formula) ≥30 mL/min;  8) International Normalized Ratio (INR) or Prothrombin Time (PT) ≤1.5 times ULN.  11. For female participants of childbearing potential, must undergo a blood pregnancy test within the first 3 days of randomization with negative results and agree to use a reliable and effective method of contraception during the trial and within 120 days of the last trial drug administration. Male patients whose partners are women of childbearing age must agree to use a reliable and effective method of contraception during the trial and within 120 days of the last trial drug administration.  12. All participants (both male and female) who are at risk of conceiving must use a contraceptive method with a failure rate of less than 1% per year throughout the treatment period and for 120 days after the last administration of the study drug (or 180 days after the last administration of study drug). |
| **Exclusion Criteria** | Participants will be excluded from the study if they meet any of the following criteria:  1. Cholangiocarcinoma (ICC), sarcomatoid hepatocellular carcinoma, and hepatic fibrolamellar carcinoma.  2. History of organ transplantation or hepatic encephalopathy.  3. Tumor burden exceeding 70% of liver volume.  4. Presence of clinically symptomatic pleural effusion, ascites, or pericardial effusion requiring drainage.  5. History of any renal disease or nephrotic syndrome.  6. History of esophageal or gastric variceal bleeding due to portal hypertension in the past 6 months; known severe (G3) varices from endoscopy within 3 months before first administration; evidence of portal hypertension (including imaging findings of splenomegaly with a longitudinal diameter over 10 cm and platelets below 100×10^9^/L), with high bleeding risk assessed by the investigators.  7. Any life-threatening bleeding event in the past 3 months, including those requiring transfusion treatment, surgery, or local therapy, ongoing medication treatment.  8. History of arterial or venous thromboembolic events in the past 6 months, including myocardial infarction, unstable angina, cerebrovascular accident or transient ischemic attack, pulmonary embolism, deep vein thrombosis, or any other serious thromboembolic disease. Exceptions include catheter-related thrombosis or superficial vein thrombosis that has stabilized after conventional anticoagulant therapy.  9. Severe bleeding tendency or coagulopathy, or are receiving thrombolytic therapy.  10. Need for long-term use of vitamin K antagonists (such as warfarin) or low-dose low molecular weight heparin (such as enoxaparin 40 mg/day) or heparin.  11. Need for long-term use of drugs that can inhibit platelet function, such as aspirin, dipyridamole, or clopidogrel.  12. Uncontrollable hypertension, with systolic blood pressure >140 mmHg or diastolic blood pressure >90 mmHg after optimal medical treatment, history of hypertensive crisis, or hypertensive encephalopathy.  13. Symptomatic congestive heart failure (New York Heart Association Class II-IV), symptomatic or poorly controlled arrhythmia, history of congenital long QT syndrome or screening corrected QTc >500 ms (calculated using Fridericia's formula).  14. History of gastrointestinal perforation and/or fistula, intestinal obstruction (including partial intestinal obstruction requiring parenteral nutrition), extensive bowel resection (partial colectomy or extensive small bowel resection with chronic diarrhea), Crohn's disease, ulcerative colitis, or long-term chronic diarrhea within the past 6 months.  15. Major surgical procedures (craniotomy, thoracotomy, or laparotomy) or unhealed wounds, ulcers, or fractures within 4 weeks prior to the first administration; tissue biopsy or other minor surgeries within 7 days prior to the first administration, except for venous catheterization for intravenous infusion.  16. History of pulmonary fibrosis, interstitial pneumonia, pneumoconiosis, drug-related pneumonia, or other severe lung diseases with significant impairment of lung function.  17. Active acute or chronic hepatitis B or C infection. Hepatitis C virus (HCV) RNA >10^3^ copies/mL; positive for both hepatitis B surface antigen (HbsAg) and anti-HCV antibodies; hepatitis B virus (HBV) DNA positive but has received antiviral treatment can be enrolled  18. Active tuberculosis, undergoing anti-tuberculosis treatment or having received anti-tuberculosis treatment within 1 year prior to the first administration.  19. Human immunodeficiency virus (HIV) infection (positive for HIV 1/2 antibodies), known syphilis infection.  20. Severe infection in active phase or poorly controlled clinically. Severe infection within 4 weeks prior to the first administration, including but not limited to hospitalization for complications due to infection, bacteremia, or severe pneumonia.  21. Active autoimmune disease requiring systemic treatment (e.g., disease-modifying drugs, corticosteroids, or immunosuppressants) within 2 years prior to the first administration; replacement therapy allowed (e.g., thyroxine, insulin, or physiological corticosteroids for adrenal or pituitary insufficiency, etc.); known primary immunodeficiency; subjects with only positive autoimmune antibodies need to be assessed by the researcher to confirm the presence of autoimmune disease.  22. Use of immunosuppressive drugs within 4 weeks prior to the first administration, excluding nasal, inhaled, or other local routes of corticosteroids or physiological doses of systemic corticosteroids (i.e., no more than 10 mg/day of prednisone or an equivalent dose of other corticosteroids), allowed for temporary use for symptoms of respiratory distress due to diseases such as asthma, chronic obstructive pulmonary disease, etc.  23. Received live attenuated vaccines within 4 weeks before the first dose or plan to receive live attenuated vaccines during the study period.  24. Received traditional Chinese medicine with anti-tumor indications or drugs with immunomodulatory effects (including thymosin, interferon, interleukin, except for local use to control pleural effusion or ascites) within 2 weeks before the first administration.  25. Uncontrolled/correctable metabolic disorders or other non-malignant organ diseases or systemic diseases or cancer-related reactions, which could lead to higher medical risks and/or uncertainty in survival evaluation.  26. Diagnosed with other malignancies within 5 years before the first administration, excluding adequately treated basal cell carcinoma of the skin, squamous cell carcinoma of the skin, and/or in situ carcinoma that has been radically resected. If diagnosed with other malignancies more than 5 years before administration, pathological or cytological diagnosis of recurrent or metastatic lesions is required.  27. Previous treatment with any anti-PD-1, anti-PD-L1/L2, anti-CTLA4 antibodies, or other immunotherapy.  28. Known allergy to sintilimab or bevacizumab or any excipients, or severe allergic reactions to other monoclonal antibodies.  29. Participation in other clinical trial treatments within 4 weeks before the first administration.  30. Pregnant or breastfeeding female patients.  31. Other acute or chronic diseases, mental illnesses, or abnormal laboratory test values that could result in increased related risks of participating in the study or administering the study drug, or interfere with the interpretation of the study results, and the patient is deemed ineligible to participate in the study by the researcher. |
| **Test drugs** | **Sintilimab Injection**  1. Specification: 100 mg/10 ml  2. Administration method: 200 mg, intravenous infusion, with a treatment cycle every 3 weeks, administered on Day 1 of each cycle.  **IBI310 (Bevacizumab Biosimilar, Recombinant Anti-VEGF Humanized Monoclonal Antibody Injection)**  1. Specification: 100mg/4ml  2. Administration method: 15mg/kg, intravenous infusion, with a treatment cycle every 3 weeks, administered on Day 1 of each cycle.  For the convenience of treatment, efforts should be made to coordinate the administration of Sintilimab and the Bevacizumab biosimilar on the same day. Sintilimab 200 mg should be given first, with an intravenous infusion lasting 30-60 minutes, followed by at least a 5-minute interval before administering the Bevacizumab biosimilar. The first intravenous infusion of the Bevacizumab biosimilar should last 90 minutes. If the first infusion is well tolerated, the time for the second infusion can be reduced to 60 minutes. If the participant also tolerates the 60-minute infusion well, then all subsequent infusions can be completed in 30 minutes.  If the Bevacizumab biosimilar is temporarily discontinued due to related toxicity, the researcher may decide whether to continue using Sintilimab alone. However, if Sintilimab is temporarily discontinued due to related toxicity, both treatments must be interrupted until the toxicity improves to baseline or at least Grade 1 according to NCI CTCAE criteria.  After stopping Bevacizumab biosimilar treatment due to surgery, Sintilimab can be used alone. |
| **Endpoints assessment** | **Safety Evaluation:**  During the study and follow-up period, the severity of adverse events will be graded according to the National Cancer Institute Common Terminology Criteria for Adverse Events (NCI CTCAE) version 5.0. Safety evaluation include:  1. The incidence, severity, and relatedness to the study drug of all Adverse Events (AEs), Treatment-Related Adverse Events (TRAEs), Serious Adverse Events (SAEs), and immune-related Adverse Events (irAEs);  2. Surgical safety assessment: Intraoperative blood loss, the incidence of post-hepatectomy liver failure (PHLF) assessed by the International Study Group of Liver Surgery (ISGLS) 2012 version, and postoperative complications graded according to the modified Clavien-Dindo system;  3. The number and proportion of participants whose treatment and surgery were delayed or discontinued due to the aforementioned adverse events.  **Efficacy Evaluation:**  1. EFS: Defined as the time from enrollment to the occurrence of disease progression, relapse, or death (whichever occurs first)  2. Conversion to Resectability Rate: Defined as the proportion of participants who undergo curative resection out of the total number of participants.  3. Pathological Response Rate: The proportion of tumor necrosis in the surgical resection specimen as demonstrated by case examination, with pathology judgment as the standard.  4. Objective Response Rate (ORR): Defined as the proportion of participants with Complete Response (CR) and Partial Response (PR) out of the total number of participants.  5. RFS: Defined as the time from curative resection to tumor recurrence or death (whichever occurs first);  6. Progression-Free Survival (PFS): Defined as the time from enrollment to the occurrence of disease progression or death (whichever occurs first)  7. Overall Survival (OS): Defined as the time from enrollment to death from any cause. EFS, ORR, RFS, and PFS are assessed by the researchers according to RECIST 1.1 criteria. |
| **Statistics** | **Sample Size:** This is an exploratory Phase Ib study, aiming to enroll a total of 30 participants. Based on the Simon two-stage design, it is anticipated that the conversion to resectability rate will be greater than 30%. A conversion to resectability rate lower than 10% will indicate the ineffectiveness of the conversion treatment plan. In the first stage, 12 participants are required; if fewer than 1 case of conversion to resectability is observed, enrollment will be terminated. If more than 1 case of conversion to resectability is observed in the first stage, the study will proceed to the second stage until a total of 30 participants are enrolled. If the total number of conversions to resectability cases across both stages is less than 5, the conversion treatment plan will be considered ineffective.  **Statistical Analysis Method:** Continuous variables will be described using means, standard deviations, medians, minimums, and maximums, while categorical variables will be described using frequencies and percentages. Kaplan-Meier estimates will be used to calculate median Event-Free Survival (mEFS), median Recurrence-Free Survival (mRFS), median Progression-Free Survival (mPFS), and median OS (mOS). |

# 1. Background

1.1 Epidemiological Background of Liver Cancer

According to GLOBOCAN 2018 statistics, in 2018, there were 840,000 new cases of liver cancer worldwide, with 780,000 deaths, making it the sixth most common cancer and the fourth leading cause of cancer death. The majority of liver cancers are Hepatocellular carcinoma (HCC). The East Asian region, especially China, Japan, and South Korea, are high-incidence areas for liver cancer, with China accounting for about 59% of the global new cases annually [1]. Epidemiological studies indicate that Hepatitis B virus (HBV) infection is the main reason for the high incidence of liver cancer in China. The diagnosis of liver cancer in China often comes late, with advanced stages at presentation, and most patients lose the opportunity for surgical resection at the time of diagnosis, leading to poor overall prognosis, with a 5-year survival rate of about 12.5% [2]. According to China's "Guidelines for the Diagnosis and Treatment of Primary Liver Cancer," for inoperable liver cancer, preoperative TACE, external radiation, and other treatments may promote tumor downstaging, thus providing some patients with the opportunity for surgical resection, and patients who undergo resection after downstaging may achieve better long-term survival outcomes [3].

1.2 Progress in Liver Cancer Immunotherapy

The advent of immune checkpoint inhibitors has brought new hope to the treatment of liver cancer. The CheckMate-040 study included 214 patients with advanced HCC treated with nivolumab, with a median progression-free survival (mPFS) of 4.0 (2.9–5.4) months, an objective response rate (ORR) of 20%, and a disease control rate (DCR) of 64% [4]. The Keynote-224 study enrolled 104 patients with advanced HCC who had failed sorafenib treatment, treated with pembrolizumab, with an mPFS of 4.9 (3.4–7.2) months, a median overall survival (mOS) of 12.9 (9.7–15.5) months; the ORR reached 17%, and DCR reached 62% [5]. Despite significant breakthroughs in the treatment of advanced liver cancer brought by immunotherapy, the efficacy of monotherapy is limited. Since angiogenesis is an important morphology and basis for tumor development, combining immunotherapy with anti-angiogenic drugs can improve the tumor microenvironment, conducive to more effective action of PD-1/PD-L1 monoclonal antibodies. Immune checkpoint inhibitors and anti-angiogenic targeted drugs have already made significant progress in cancers such as lung cancer.

IMbrave150 is a study evaluating atezolizumab combined with bevacizumab as a first-line treatment for unresectable HCC in patients who have not previously received systemic treatment, enrolling 501 patients, randomized in a 2:1 ratio to receive atezolizumab combined with bevacizumab or sorafenib. The results of IMbrave150, presented at the 2019 European Society for Medical Oncology (ESMO) Asia Conference, showed that compared to sorafenib, the treatment group of atezolizumab (1200 mg IV Q3W) combined with bevacizumab (15mg/kg IV Q3W) reduced the risk of death by 42% (HR=0.58, 95% CI:0.42-0.79, p=0.0006) and reduced the risk of disease progression by 41% (HR=0.59, 95% CI:0.47-0.76, p<0.0001), with mPFS of 6.8 vs 4.3 months. The complete response rate (CR) was 10% vs 2% (mRECIST), and the ORR was 33% vs 13% (mRECIST), respectively [6].

According to the results of the IMbrave150 China subgroup announced at the 2020 European Association for the Study of the Liver (EASL) Liver Cancer Summit, which included 190 patients (ITT), the overall results were consistent with the global outcomes. Compared to sorafenib, the treatment group receiving atezolizumab combined with bevacizumab reduced the risk of death by 56% (HR=0.44, 95% CI:0.25-0.7), with 6-month PFS rates of 48% vs 31%; reduced the risk of disease progression by 40% (HR=0.60, 95%CI:0.40-0.90), with 6-month OS rates of 87% vs 64%; CR rates of 13% vs 0 (mRECIST), and ORR rates of 30% vs 8% (mRECIST)[7]. In the IMbrave150 study, 15.6% (78/501) of the patients were in BCLC stage B, and subgroup analysis showed no difference in OS between the two groups for BCLC B stage patients (HR=1.09, 95%CI:0.33-3.53); however, PFS was better in the atezolizumab combined with bevacizumab group compared to the sorafenib group (HR=0.65, 95%CI:0.33-1.30).

The 2020.V1 edition of the NCCN Clinical Practice Guidelines in Oncology: Hepatobiliary Cancers has already recommended atezolizumab combined with bevacizumab for first-line treatment of HCC. In the IMbrave150 study, the ORR (mRECIST) for atezolizumab combined with bevacizumab was 33%, with CR (mRECIST) of 10%, significantly higher than the 13% and 2% with sorafenib treatment, respectively. The REFLECT study was a non-inferiority study comparing lenvatinib and sorafenib for first-line treatment of unresectable advanced liver cancer [8]. In the REFLECT study, ORR (mRECIST) for lenvatinib vs sorafenib was 24.3% and 9.4%, respectively, with CR (mRECIST) of 1.3% vs 0.4%. The ORR and CR for atezolizumab combined with bevacizumab were significantly higher than those for sorafenib and lenvatinib, suggesting that the benefit in ORR could bring hope for potentially resectable HCC to undergo curative resection.

1.3 Sintilimab

### 1.3.1 Mechanism of Action of Sintilimab

Sintilimab (development code: IBI308) is a recombinant fully human IgG4 monoclonal antibody against PD-1, developed by Innovent Biologics (Suzhou) Co., Ltd. It specifically binds to the PD-1 molecule on the surface of T lymphocytes, thereby blocking the PD-1/PD-L1 pathway that leads to tumor immune tolerance, reactivating the anti-tumor activity of T lymphocytes. Sintilimab targets the same molecule as nivolumab and pembrolizumab but has a different amino acid sequence. Multiple preclinical in vitro studies have verified the effect of Sintilimab in blocking the PD-1 pathway. Completed preclinical pharmacodynamics, animal pharmacokinetics, and toxicology studies have shown that Sintilimab has a clear target, reliable cell line origin, and good drug stability, and has shown good activity in completed preclinical studies.

### 1.3.2 Pharmacokinetics and Pharmacodynamics of Sintilimab

In September 2016, a phase Ia dose-escalation trial of Sintilimab (study code CIBI308A101-1a) was initiated. The phase Ia enrolled patients with advanced solid tumors who had failed standard treatments, with dose increases following the classic "3+3" design, evaluating 4 dose levels (1mg/kg, 3 mg/kg, 200mg, and 10mg/kg). After completing the 1mg/kg dose group, participants were randomly assigned 1:1 to independently evaluate the 3mg/kg and 200mg dose groups. The DLT observation period was 28 days after the first dose in each dose group, and participants who completed the DLT observation period continued treatment with Sintilimab every two weeks (1mg/kg, 3mg/kg, and 10mg/kg) or every three weeks (200mg) until disease progression, intolerable toxicity, withdrawal of consent, or other reasons to stop study treatment occurred (whichever came first).

The pharmacokinetic study of Sintilimab in advanced solid tumor patients showed that after intravenous administration, blood drug concentrations gradually increased, reaching Cmax after stopping the drug, then slowly decreased. Within the 1-10mg/kg dose range, the exposure of Sintilimab in the body increased nearly proportionally with the dose, indicating linear kinetics. The elimination half-life (Geo.Mean[CV%]) of Sintilimab after a single dose in solid tumor patients was 14.4[28.9%] days, with a clearance rate of 11.5[42.5%] mL/h, steady-state distribution volume of 5.43[34.4%] L, and apparent distribution volume of 5.77[33.2%] L, similar to the PK characteristics of other marketed anti-PD-1 antibodies (nivolumab and pembrolizumab).

In the pharmacodynamic study of advanced solid tumor patients in phase Ia, a single dose of 1mg/kg (N=3) of Sintilimab rapidly (within 24 hours) saturated the occupation (average ≥95%) of PD-1 receptors on the surface of CD3+T cells in the peripheral blood of solid tumor patients and maintained the occupation level during the study period (28 days) with decreasing concentration and through continuous multiple-dose treatment. PD-1 occupation results for the 3mg/kg (N=3), 200mg (N=3), and 10mg/kg (N=3) dose groups were similar to those of the 1mg/kg dose group, indicating no dose and concentration dependence of PD-1 receptor occupation levels within the 1-10 mg/kg dose range. Based on early pharmacokinetic and pharmacodynamic results, as well as acceptable safety events and considering potential individual differences, the subsequent study dose of Sintilimab was set at 200mg Q3W.

### 1.3.3 Clinical Study Results of Sintilimab in Liver Cancer Patients

The CIBI308A101-Ib study is a multicenter, phase Ib clinical trial conducted in China among patients with advanced solid tumors, where Cohort B primarily included patients with hepatobiliary and pancreatic malignancies who failed or were intolerant to first-line therapy. Out of 36 patients who received sintilimab monotherapy and had radiological assessments, there was 1 case of complete response (CR), 4 cases of partial response (PR), 9 cases of stable disease (SD), with an objective response rate (ORR) of 13.9%, and a disease control rate (DCR) of 38.9%. The safety and tolerability in patients were good.

Currently, a phase II/III study (ORIENT-32, NCT03794440) of sintilimab combined with a bevacizumab biosimilar versus sorafenib for first-line treatment of advanced hepatocellular carcinoma is ongoing. The study plans to enroll 566 participants, randomized in a 2:1 ratio to the experimental group or control group, with the primary endpoint being overall survival. Enrollment has been completed.

Based on the results of a multicenter, single-arm, phase II clinical study (ORIENT-1) of sintilimab in recurrent or refractory classical Hodgkin lymphoma, sintilimab was officially approved by the National Medical Products Administration (NMPA) on December 24, 2018, for the treatment of classical Hodgkin lymphoma patients who have received at least two lines of systemic chemotherapy.

As of October 16, 2018, a total of 540 cancer patients had received sintilimab treatment in 5 studies, showing an overall safety profile similar to other approved anti-PD-1 monoclonal antibodies internationally. The incidence of all-grade adverse events (AEs) in the 540 patients treated with sintilimab was 86.1%, with ≥10% incidence AEs including fever, anemia, increased aspartate aminotransferase (AST), increased alanine aminotransferase (ALT), fatigue, and decreased white blood cell count. The incidence of grade 3 or higher AEs was 30.6%, with ≥1% incidence including pneumonia, anemia, increased lipase, decreased platelets, neutropenia, hyponatremia, increased γ-glutamyltransferase (γ-GT), infectious pneumonia, upper gastrointestinal bleeding, and decreased lymphocytes.

## 1.4 IBI310 (Bevacizumab Biosimilar)

### 1.4.1 Mechanism of Action of Bevacizumab Biosimilar

The bevacizumab biosimilar (IBI305) is a recombinant anti-VEGF humanized monoclonal antibody injection developed by Innovent Biologics as a biosimilar. It specifically binds to human VEGFA and inhibits its interaction with VEGFR1 and VEGFR2, blocking signaling pathways such as PI3K-Akt/PKB and Ras-Raf-MEK-ERK, inhibiting endothelial cell growth, proliferation, and migration, as well as angiogenesis, reducing vascular permeability, and thereby inhibiting tumor cell proliferation and metastasis, and inducing tumor cell apoptosis. Studies in pharmacology, pharmacodynamics, and animal pharmacokinetics have shown that the bevacizumab biosimilar has high similarity to bevacizumab, suggesting potential similar clinical efficacy and safety, thus warranting clinical trials.

### 1.4.2 Clinical Study Results of Bevacizumab Biosimilar

In a clinical phase I study of 100 patients randomized 1:1 to either the bevacizumab biosimilar group or the bevacizumab group, the pharmacokinetic profiles of the biosimilar and bevacizumab were similar, with no significant differences in PK parameters between the two groups. The bioequivalence analysis showed that the main PK parameters (AUC0-∞ and Cmax) of the bevacizumab biosimilar group fell within the acceptable equivalence range (80%-125%), indicating bioequivalence to bevacizumab.

The most common treatment-emergent adverse events (TEAEs) related to the bevacizumab biosimilar included hypertriglyceridemia, increased ALT, hyperglycemia, increased AST, increased white blood cell count, increased neutrophil count, decreased white blood cell count, positive urinary red blood cells, sinus bradycardia, decreased neutrophil count, headache, and insomnia. The severity of hypertriglyceridemia, increased AST, and decreased neutrophil count was grade 3.

Preclinical pharmacology, pharmacodynamics, pharmacokinetics, and clinical pharmacokinetics results indicate that the bevacizumab biosimilar has high similarity to bevacizumab injection, thus expected to have similar clinical efficacy and safety, allowing for clinical trial conduction based on bevacizumab injection data.

In a randomized, double-blind phase III study comparing the efficacy and safety of the bevacizumab biosimilar or bevacizumab (Avastin) combined with paclitaxel/carboplatin as first-line treatment for advanced non-squamous non-small cell lung cancer (NSCLC), 450 patients were randomized 1:1 into the bevacizumab biosimilar group or bevacizumab group. The primary endpoint ORR was 44.3% in the biosimilar group and 46.4% in the bevacizumab group, with an ORR ratio of 1.01 (90% CI: 0.803-1.135), within the pre-specified equivalence range (0.75-1.33); secondary endpoint PFS was 7.64 months for the biosimilar group and 7.77 months for the bevacizumab group (HR 1.00, 95% CI:0.808-1.237, p=0.9987), confirming the clinical equivalence hypothesis. There was no significant difference in AE incidence between the groups, with SAE incidence being 33.5% and 37.6%, and grade 3 or higher AE incidence being 84.4% and 89.8% (p=0.085) respectively. PK sampling in 76 and 75 participants respectively showed consistent group drug exposure and low and not significantly different rates of anti-drug antibodies and neutralizing antibodies between groups. This study validated the similarity in clinical efficacy, safety, population pharmacokinetics, and immunogenicity between the bevacizumab biosimilar and bevacizumab in first-line NSCLC patients [9]. The bevacizumab biosimilar has submitted a marketing application to the NMPA and is pending approval.

## 1.5 Risk/Benefit Assessment

Considering the mechanism of action of sintilimab and the clinical safety information of similar mechanism products, adverse events likely to occur during clinical trials are mainly various immune-related inflammations, such as pneumonia, colitis, hepatitis, renal dysfunction, and endocrine system inflammations. Despite the high incidence of adverse reactions with anti-PD-1 monoclonal antibody drugs, they are generally well-tolerated, with only a small portion of patients discontinuing medication due to adverse reactions, and most adverse reactions can be managed with treatment. Due to the variable early symptoms of immune-related adverse reactions, researchers should pay special attention to the early symptoms and signs of various immune-related reactions in clinical studies, make timely and correct judgments, adjust doses, and provide effective treatment to reduce the risk of drug use in patients, while excluding patients with autoimmune diseases to avoid exacerbation of pre-existing conditions due to immune system activation.

The bevacizumab biosimilar, a VEGF humanized monoclonal antibody developed in reference to bevacizumab, underwent comprehensive comparison with bevacizumab throughout its development, meeting the national regulations for biosimilars. With identical amino acid sequences and highly similar pharmacological, pharmacodynamic, preclinical toxicological characteristics, clinical pharmacokinetics, and safety profiles, the bevacizumab injection has been validated for the treatment of various tumors over more than 10 years on the market, becoming one of the most commonly used anti-cancer drugs. The most common adverse reactions to bevacizumab include hypertension, fatigue, diarrhea, and abdominal pain. Thus, the bevacizumab biosimilar is expected to have similar safety features to bevacizumab and can be managed using the same safety measures.

In the IMbrave150 study, the atezolizumab combined with bevacizumab group experienced grade 3-4 adverse events in 57% of patients, 36% of which were treatment-related; in patients receiving sorafenib treatment, 55% experienced grade 3-4 adverse events, 46% of which were treatment-related. The >10% adverse events in the atezolizumab combined with bevacizumab group included hypertension, proteinuria, diarrhea, appetite loss, fever, increased ALT, abdominal pain, infusion-related reactions, etc., with generally good tolerability and manageable toxicity; no new safety issues were found beyond the known safety events of atezolizumab and bevacizumab monotherapy [6].

Furthermore, angiogenesis is important for wound healing, so bevacizumab also increases the risk of delayed wound healing, which should be considered in perioperative care. Bevacizumab neoadjuvant therapy has been used in triple-negative breast cancer, NSCLC, gastroesophageal adenocarcinoma, rectal cancer, and surgery for colorectal cancer liver metastases, accumulating extensive experience.

In the GBG44 study of neoadjuvant therapy for triple-negative breast cancer, 1948 patients were randomized 1:1 into chemotherapy alone or chemotherapy combined with bevacizumab groups. In this study, the average interval between surgery and the last dose of bevacizumab was 34 days.

In the chemotherapy group, 962 patients completed surgery, and in the chemotherapy combined with bevacizumab group, 954 patients completed surgery. Among 743 patients assessable for postoperative complications (38.1%, 349 in the chemotherapy group, 394 in the chemotherapy combined with bevacizumab group), 38 cases (10.9%) in the chemotherapy group and 59 cases (15.0%) in the chemotherapy combined with bevacizumab group experienced postoperative complications, with no significant difference between the two groups (p=0.103). Specific complications such as bleeding/hematoma, wound infection/abscess, and delayed wound healing necrosis also showed no significant differences between the two groups[10].

In a neoadjuvant study of chemotherapy combined with bevacizumab for NSCLC, patients received 4 cycles of docetaxel plus cisplatin and 3 cycles of bevacizumab treatment before surgery, with the last pre-surgical treatment regimen not including bevacizumab. Bevacizumab was well tolerated in this study, and the incidence of perioperative complications, except for gastrointestinal complications, did not significantly increase compared to other neoadjuvant chemotherapy studies without bevacizumab. Individual cases of gastrointestinal bleeding and bronchopleural fistula in the study might be related to bevacizumab, with adverse reaction levels at grade 3[11].

According to the preliminary safety report of a multicenter phase II ST03 study comparing ECX regimen (epirubicin+cisplatin+capecitabine) with or without bevacizumab for preoperative treatment of gastroesophageal adenocarcinoma, preoperative use of bevacizumab did not increase surgical risk. The study enrolled 200 patients, randomized 1:1 into two groups, with an average interval of 56-63 days between surgery and the last dose of bevacizumab. Gastrointestinal perforation, cardiac events, and venous thrombotic events were uncommon overall in the study, with no significant differences in wound delayed healing, anastomotic fistula, and gastrointestinal bleeding rates between the two groups[12].

N-SOG 03 is a phase II single-arm multicenter study of neoadjuvant therapy with capecitabine + oxaliplatin combined with bevacizumab for patients with low-risk colorectal cancer. The study enrolled 32 patients, 30 of whom underwent surgical treatment. The average interval between the last dose of bevacizumab and surgery was 62 days. Postoperative complications occurred in 13 patients (43%), including anastomotic fistula in 5 cases, wound infection in 7 cases, pelvic infection in 3 cases, and intestinal obstruction in 6 cases. One patient died due to rectal perforation requiring emergency laparotomy and R2 resection. Of the 18 patients who underwent rectal cancer sphincter-preserving surgery, 5 (27.8%) experienced anastomotic fistula. The high incidence of surgical complications in this study suggests a significant association with bevacizumab[13].

In contrast, a phase II single-arm multicenter study of neoadjuvant therapy with capecitabine+oxaliplatin combined with bevacizumab for patients with high-risk colorectal cancer showed good safety of neoadjuvant therapy including bevacizumab. The study included 25 patients, 23 of whom underwent surgical treatment. The interval between the last dose of bevacizumab and surgery ranged from 42-77 days. Six patients (26.1%) experienced postoperative complications, including 4 cases of wound infection, 1 case of anastomotic fistula, 1 case of intestinal obstruction, and 1 case requiring further surgery for bleeding[14].

Kesmodel et al. retrospectively compared the complications of neoadjuvant chemotherapy with and without bevacizumab in colorectal cancer liver metastases, with an average time of 58 days (range: 31-117 days) from the last dose of bevacizumab to surgery. The incidence of postoperative complications was 43% (19/44) in the chemotherapy group and 49% (40/81) in the chemotherapy combined with bevacizumab group, with no significant difference between the groups. Multivariate regression analysis indicated that low albumin levels and extra-hepatic surgery increased the incidence of complications (p-values 0.035 and 0.023, respectively), and low albumin levels also increased hepatobiliary complications (p-value=0.016). Most patients with postoperative complications experienced only 1-2 complications, mostly grade 1-2. Wound-related complications were the most common, occurring in 27% of patients, mostly minor and manageable with local wound care or antibiotics. Four patients (1 in the chemotherapy group and 3 in the chemotherapy combined with bevacizumab group) experienced superficial wound dehiscence, treated with negative pressure wound therapy. Two patients (in the chemotherapy combined with bevacizumab group) required reoperation for wound dehiscence and infection[15].

The NCCN Guidelines for Colorectal Cancer 2020.V1 recommend an interval of at least 6 weeks between surgery and the last dose of bevacizumab, with bevacizumab therapy resuming 6-8 weeks postoperatively[15].

Tamandl et al. conducted a case-control study on neoadjuvant therapy before surgery for colorectal cancer liver metastases, with 102 cases in the trial group (chemotherapy combined with bevacizumab) and 112 cases in the control group (chemotherapy group), with an average time of 37 days (range: 17-99 days) from the last dose of bevacizumab to surgery. The incidence of postoperative complications was 44% and 34% (p=0.216), respectively, with no significant difference between the groups. Multivariate regression analysis showed that older age, low albumin levels, resection of more than 3 liver segments, and simultaneous intestinal resection surgery were associated with an increased incidence of complications[16].

The safety of neoadjuvant therapy with chemotherapy combined with bevacizumab for elective surgery in colorectal cancer liver metastases is considered good, with a widely accepted interval of 5-8 weeks between the last dose of bevacizumab and surgery[13].

Based on the analysis, stopping bevacizumab biosimilar 6-8 weeks before surgery for primary liver cancer minimally impacts the risk of bleeding during surgery, and bevacizumab biosimilar treatment can be resumed 5-8 weeks postoperatively. It is anticipated that the combination of sintilimab with bevacizumab biosimilar may have controllable toxic reactions and potentially synergistic antitumor effects, potentially benefiting patients with HCC undergoing transformative treatment.

This study is a prospective, single-arm exploratory study to evaluate the safety and efficacy of sintilimab combined with bevacizumab biosimilar in patients with potentially resectable intermediate-stage HCC who have not previously received HCC treatment.

**References**

1. Chen W, Zheng R, Baade PD, et al. Cancer statistics in China, 2015. CA Cancer J Clin. 2016;66(2):115- 132.
2. Zeng H, Chen W, Zheng R, et al. Changing cancer survival in China during 2003- 15: a pooled analysis of 17 population-based cancer registries. Lancet Glob Health. 2018;6(5): e555-e567.
3. National Health Commission of the People's Republic of China. (2019). Guidelines for the Diagnosis and Treatment of Primary Liver Cancer.
4. Kudo M, Matilla A, Santoro A, et al. Checkmate-040: Nivolumab in patients with advanced hepatocellular carcinoma and Child-Pugh B status. J Clin Oncol. 2019; 37(4suppl)327.
5. Zhu AX, Finn RS, Edeline J, et al. Pembrolizumab in patients with advanced hepatocellular carcinoma previously treated with sorafenib (KEYNOTE-224): a non-randomised, open-label phase 2 trial. Lancet Oncol. 2018;19(7):940-952.
6. Cheng AL, Qin S, Ikeda M. IMbrave150：efficacy and safety results from a phase 3 study evaluating atezolizumab plus bevacizumab vs sorafenib as first treatment for patients with unresectable Hepatocellular Carcinoma[C]. 2019 ESMO Asia: LBA03.
7. Qin S, Galle PR, Ren ZG, et al. Efficacy and safety of atezolizumab plus bevacizumab vs sorafenib in Chinese patients with unresectable HCC in the Phase Ⅲ IMbrave150 study. Liver Cancer Summit 2020; OP02-02.
8. Kudo M, Finn RS, Qin S, et al. Lenvatinib versus sorafenib in first-line treatment of patients with unresectable hepatocellular carcinoma: a randomised phase 3 non-inferiority trial. Lancet. 2018;391(10126):1163- 1173.
9. Yang Y, Wu B, Huang L, et al. Biosimilar candidate IBI305 plus paclitaxel/carboplatin for the treatment of non-squamous non-small cell lung cancer. Transl Lung Cancer Res. 2019;8(6):989-999.
10. Gerber B, von Minckwitz G, Eidtmann H, et al. Surgical outcome after neoadjuvant chemotherapy and bevacizumab: results from the GeparQuinto study (GBG 44). Ann Surg Oncol. 2014;21(8):2517-2524.
11. Chaft JE, Rusch V, Ginsberg MS, et al. Phase II trial of neoadjuvant bevacizumab plus chemotherapy and adjuvant bevacizumab in patients with resectable nonsquamous non-small-cell lung cancers. J Thorac Oncol. 2013;8(8):1084- 1090.
12. Okines AF, Langley RE, Thompson LC, et al. Bevacizumab with peri-operative epirubicin, cisplatin and capecitabine (ECX) in localised gastro-oesophageal adenocarcinoma: a safety report. Ann Oncol. 2013;24(3):702-709.
13. Uehara K, Hiramatsu K, Maeda A, et al. Neoadjuvant oxaliplatin and capecitabine and bevacizumab without radiotherapy for poor-risk rectal cancer: N-SOG 03 Phase II trial. Jpn J Clin Oncol. 2013;43(10):964-971.
14. Hasegawa J, Nishimura J, Mizushima T, et al. Neoadjuvant capecitabine and oxaliplatin (XELOX) combined with bevacizumab for high-risk localized rectal cancer. Cancer Chemother Pharmacol. 2014;73(5):1079- 1087.
15. Kesmodel SB, Ellis LM, Lin E, et al. Preoperative bevacizumab does not significantly increase postoperative complication rates in patients undergoing hepatic surgery for colorectal cancer liver metastases. J Clin Oncol. 2008;26(32):5254-5260.
16. Tamandl D, Gruenberger B, Klinger M, et al. Liver resection remains a safe procedure after neoadjuvant chemotherapy including bevacizumab: a case-controlled study. Ann Surg. 2010;252(1):124- 130.

# 2. Study objectives:

## 2.1 Primary Objectives:

- To evaluate the safety and efficacy of sintilimab/bev followed by resection in patients with intermediate-stage HCC.
- To assess Event-Free Survival (EFS) as evaluated by RECIST 1.1.

## 2.2 Secondary Objectives:

- To determine the conversion to resectability rate (R0 resection rate).
  - To assess the pathological response rate.
  - To assess the Objective Response Rate (ORR) as evaluated by RECIST 1.1.
  - To assess Recurrence-Free Survival (RFS) in participants who receive surgical resection, as per RECIST 1.1.
  - To assess Progression-Free Survival (PFS) in participants who do not receive surgical resection, as per RECIST 1.1.
  - To assess OS in participants who receive surgical resection.
  - To assess OS in participants who do not receive surgical resection.
  - To assess OS in all participants.

## 2.3 Exploratory Objectives:

To explore the value of blood biomarkers in predicting treatment efficacy and toxic reactions. Peripheral blood (10 mL) will be collected from subjects within 14 days prior to the first medication and at the time of surgery or disease progression, and sent to Pu'enhaihui Medical Laboratory Co., Ltd. in Shanghai for testing

(registered location: Rooms 501, 502, 503, 601, 602, Building 7, No. 1158 Center Road, Songjiang High-tech Industrial Park, Caohejing Development Zone, Shanghai; ZIP code: 201615; Mailing address: 5th Floor, Building No. 7, No. 1158 Center Road, Songjiang District, Shanghai),

for the following tests:

1. Epi CGP ctDNA (Pan-cancer Genomic Profiling - ctDNA): This test detects single nucleotide variations (SNVs), small fragment insertions and deletions (InDels), copy number variations (CNVs), and structural variations (SVs) in cancer-related genes. It analyzes mutations in relevant pathways and studies mutations significantly correlated with treatment efficacy, blood tumor mutational burden (bTMB), or microsatellite instability (MSI), thereby discovering biomarkers that can predict treatment efficacy.
2. T-cell Receptor (TCR) Diversity Sequencing: By sequencing, various combinations of TCR clones are obtained. Diversity indices such as TCR evenness and convergence are calculated, and their correlations with treatment efficacy and immune-related toxic reactions are analyzed.

# 3. Study design

## 3.1 Overall study design

This study is a single-arm, prospective study targeting patients with initially treated, potentially resectable intermediate-stage HCC (CNLC-IIa and IIb) to explore the safety and efficacy of combined treatment with Sintilimab and a biosimilar of Bevacizumab (IBI310) in the conversion therapy of HCC.

The plan is to enroll a total of 30 patients with potentially resectable intermediate-stage HCC to receive first-line treatment with 200 mg IV Sintilimab on day 1 every 3 weeks combined with 15 mg/kg IV biosimilar of Bevacizumab on day 1 every 3 weeks. Within the first 6 months after starting the medication, efficacy and surgical resection assessment will be conducted every 6 weeks according to RECIST 1.1 criteria (evaluated by researchers at each center), thereafter switching to evaluations every 9 weeks. Patients deemed eligible for curative surgery by the researchers will undergo surgical treatment. For those undergoing surgery, adjuvant therapy post-surgery will not exceed 12 months; for those not undergoing surgery, treatment will continue until disease progression, occurrence of intolerable toxic reactions, initiation of new anti-tumor treatment, withdrawal of informed consent, loss to follow-up, death, or any other situation requiring termination of treatment as specified in the protocol, whichever occurs first, up to a maximum of 24 months.

Based on the Simon's two-stage design, the expected conversion resection rate is greater than 30%. If the conversion resection rate is below 10%, the conversion therapy regimen is considered ineffective. In the first stage, 12 patients will be enrolled. If fewer than 1 patient achieves conversion resection, enrollment will be terminated; if more than 1 patient achieves conversion resection in the first stage, the second stage will proceed until a total of 30 patients are enrolled. If fewer than 5 patients achieve conversion resection across both stages, the conversion therapy regimen will be considered ineffective.

# 4.Study population

## 4.1 Inclusion criteria

Participants eligible for this study must meet all the following criteria:

1. Able to provide informed consent and willing to sign an approved consent form before any trial-related procedures are conducted.

2. Male or female, aged ≥18 and ≤75 years.

3.Diagnosed with hepatocellular carcinoma (HCC) through histology/cytology or meets the clinical diagnostic standards of the 2019 edition of the "Guidelines for the Diagnosis and Treatment of Primary Liver Cancer" issued by the National Health Commission of China.

4. Intermediate-stage HCC (CNLC-IIa and IIb) deemed potentially resectable upon assessment by the researcher.

5. No prior treatment for HCC.

6. Child-Pugh class A.

7. Eastern Cooperative Oncology Group Performance Status (ECOG PS) score of 0-1.

8. Expected survival time of more than 6 months.

9. At least 1 measurable lesion according to RECIST 1.1 criteria.

10. Adequate bone marrow and organ function, with the participant needing to meet the following laboratory criteria:

1) Absolute Neutrophil Count (ANC) ≥1.0x10^9/L without the use of granulocyte colony-stimulating factor within the last 14 days;

2) Platelets ≥75x10^9/L without transfusion within the last 14 days;

3) Hemoglobin ≥9g/dL without transfusion or the use of erythropoiesis-stimulating agents within the last 14 days;

4) Total bilirubin ≤2.0 times the upper limit of normal (ULN);

5) Albumin ≥2.8g/dL;

6) Aspartate aminotransferase (AST) and Alanine aminotransferase (ALT) within ≤5 times ULN;

7) Creatinine ≤1.5 times ULN and creatinine clearance (calculated using the Cockcroft-Gault formula) ≥30 ml/min;

8) Good coagulation function, defined as International Normalized Ratio (INR) or Prothrombin Time (PT) ≤1.5 times ULN.

11. For female participants of childbearing potential, must undergo a blood pregnancy test within the first 3 days of randomization with negative results and agree to use a reliable and effective method of contraception during the trial and within 120 days of the last trial drug administration. Male patients whose partners are women of childbearing age must agree to use a reliable and effective method of contraception during the trial and within 120 days of the last trial drug administration.

12. All participants (both male and female) who are at risk of conceiving must use a contraceptive method with a failure rate of less than 1% per year throughout the treatment period and for 120 days after the last administration of the study drug (or 180 days after the last administration of chemotherapy).

## 4.2 Exclusion criteria

Participants will be excluded from the study if they meet any of the following criteria:

1. Known as cholangiocarcinoma (ICC) or mixed hepatocellular carcinoma, sarcomatoid hepatocellular carcinoma, and hepatic fibrolamellar carcinoma.

2. History of organ transplantation or hepatic encephalopathy.

3. Tumor burden exceeding 70% of liver volume.

4. Presence of clinically symptomatic pleural effusion, ascites, or pericardial effusion requiring drainage.

5. History of any renal disease or nephrotic syndrome.

6. History of esophageal or gastric variceal bleeding due to portal hypertension in the past 6 months; known severe (G3) varices from endoscopy within 3 months before first administration; evidence of portal hypertension (including imaging findings of splenomegaly with a longitudinal diameter over 10cm and platelets below 100×10^9/L), with high bleeding risk assessed by the researcher.

7. Any life-threatening bleeding event in the past 3 months, including those requiring transfusion treatment, surgery, or local therapy, ongoing medication treatment.

8. History of arterial or venous thromboembolic events in the past 6 months, including myocardial infarction, unstable angina, cerebrovascular accident or transient ischemic attack, pulmonary embolism, deep vein thrombosis, or any other serious thromboembolic disease. Exceptions include catheter-related thrombosis or superficial vein thrombosis that has stabilized after conventional anticoagulant therapy.

9. Severe bleeding tendency or coagulopathy, or are receiving thrombolytic therapy.

10. Need for long-term use of vitamin K antagonists (such as warfarin) or low-dose low molecular weight heparin (such as enoxaparin 40 mg/day) or heparin.

11. Need for long-term use of drugs that can inhibit platelet function, such as aspirin, dipyridamole, or clopidogrel.

12. Uncontrollable hypertension, with systolic blood pressure >140mmHg or diastolic blood pressure >90mmHg after optimal medical treatment, history of hypertensive crisis, or hypertensive encephalopathy.

13. Symptomatic congestive heart failure (New York Heart Association Class II-IV), symptomatic or poorly controlled arrhythmia, history of congenital long QT syndrome or screening corrected QTc >500ms (calculated using Fridericia's formula).

14. History of gastrointestinal perforation and/or fistula, intestinal obstruction (including partial intestinal obstruction requiring parenteral nutrition), extensive bowel resection (partial colectomy or extensive small bowel resection with chronic diarrhea), Crohn's disease, ulcerative colitis, or long-term chronic diarrhea within the past 6 months.

15. Major surgical procedures (craniotomy, thoracotomy, or laparotomy) or unhealed wounds, ulcers, or fractures within 4 weeks prior to the first administration; tissue biopsy or other minor surgeries within 7 days prior to the first administration, except for venous catheterization for intravenous infusion.

16. History of pulmonary fibrosis, interstitial pneumonia, pneumoconiosis, drug-related pneumonia, or other severe lung diseases with significant impairment of lung function, both past and present.

17. Active acute or chronic hepatitis B or C infection. Hepatitis C virus (HCV) RNA >10^3 copies/ml; positive for both hepatitis B surface antigen (HbsAg) and anti-HCV antibodies; hepatitis B virus (HBV) DNA positive but has received antiviral treatment may enter the study.

18. Active tuberculosis (TB), undergoing anti-tuberculosis treatment or having received anti-tuberculosis treatment within 1 year prior to the first administration.

19. Human immunodeficiency virus (HIV) infection (positive for HIV 1/2 antibodies), known syphilis infection.

20. Severe infection in active phase or poorly controlled clinically. Severe infection within 4 weeks prior to the first administration, including but not limited to hospitalization for complications due to infection, bacteremia, or severe pneumonia.

21. Active autoimmune disease requiring systemic treatment (e.g., disease-modifying drugs, corticosteroids, or immunosuppressants) within 2 years prior to the first administration; replacement therapy allowed (e.g., thyroxine, insulin, or physiological corticosteroids for adrenal or pituitary insufficiency, etc.); known primary immunodeficiency; subjects with only positive autoimmune antibodies need to be assessed by the researcher to confirm the presence of autoimmune disease.

22. Use of immunosuppressive drugs within 4 weeks prior to the first administration, excluding nasal, inhaled, or other local routes of corticosteroids or physiological doses of systemic corticosteroids (i.e., no more than 10mg/day of prednisone or an equivalent dose of other corticosteroids), allowed for temporary use for symptoms of respiratory distress due to diseases such as asthma, chronic obstructive pulmonary disease, etc.

23. Received live attenuated vaccines within 4 weeks before the first dose or plan to receive live attenuated vaccines during the study period.

24. Received traditional Chinese medicine with anti-tumor indications or drugs with immunomodulatory effects (including thymosin, interferon, interleukin, except for local use to control pleural effusion or ascites) within 2 weeks before the first administration.

25. Uncontrolled/correctable metabolic disorders or other non-malignant organ diseases or systemic diseases or cancer-related reactions, which could lead to higher medical risks and/or uncertainty in survival evaluation.

26. Diagnosed with other malignancies within 5 years before the first administration, excluding adequately treated basal cell carcinoma of the skin, squamous cell carcinoma of the skin, and/or in situ carcinoma that has been radically resected. If diagnosed with other malignancies more than 5 years before administration, pathological or cytological diagnosis of recurrent or metastatic lesions is required.

27. Previous treatment with any anti-PD-1, anti-PD-L1/L2, anti-CTLA4 antibodies, or other immunotherapy.

28. Known allergy to sintilimab, bevacizumab agents, and excipients, or severe allergic reactions to other monoclonal antibodies in the past.

29. Participation in other clinical trial treatments within 4 weeks before the first administration.

30. Pregnant or breastfeeding female patients.

31. Other acute or chronic diseases, mental illnesses, or abnormal laboratory test values that could result in increased related risks of participating in the study or administering the study drug, or interfere with the interpretation of the study results, and the patient is deemed ineligible to participate in the study by the researcher.

## 4.3 Restrictions During the Study Period

### 4.3.1 Pregnancy

It is known that human IgG1 and IgG4 can cross the placental barrier. Medication during pregnancy is not recommended. Pregnant women are not eligible for this study.

### 4.3.2 Reproductive Age

Female participants of reproductive potential who are sexually active with a non-sterilized male partner, as well as non-sterilized male participants who are sexually active with a female partner of reproductive potential, must use one of the acceptable and effective methods of contraception listed in Table 1 from the screening period until 180 days after the last dose of medication. Participants and their partners must discuss with a responsible physician the discontinuation of contraception after this time point. Periodic abstinence, the rhythm method, and withdrawal are not acceptable methods of contraception. Women of reproductive potential are defined as those who have experienced menarche, have not undergone sterilization (i.e., bilateral tubal ligation, bilateral oophorectomy, or hysterectomy), and have not yet reached menopause.

Table 1: Effective Methods of Contraception (at least one method must be used)

| Barrier Methods | Intrauterine Device (IUD) Methods | Hormonal Methods |
| --- | --- | --- |
| Male Condoms with Spermicide Cervical Cap with Spermicide  Diaphragm with Spermicide | Copper Intrauterine Device (Cu-IUD)  Levonorgestrel-Releasing Intrauterine Device (LNG-IUD) ^a^  Levonorgestrel-Releasing Intrauterine System (e.g., Mirena®)^a^ | Implants  Hormonal Contraceptive Injections  Combined Oral Contraceptive Pills  Low-Dose Oral Contraceptive Pills  Contraceptive Patch |

a ．This is also considered a hormonal method.

A woman is considered to be in menopause after 12 months of amenorrhea in the absence of other biological or physiological causes.

Based on age requirements:

- Women under 50 years of age are considered postmenopausal if they have been amenorrheic for 12 months or more following cessation of exogenous hormone treatments and their levels of luteinizing hormone and follicle-stimulating hormone are within the postmenopausal range.

- Women aged 50 years or older are considered postmenopausal if they have been amenorrheic for 12 months or more following cessation of all exogenous hormone treatments, have had radiation-induced oophorectomy with the last menstruation occurring more than 1 year ago, have had chemotherapy-induced amenorrhea with more than 1 year since the last menstruation, or have undergone surgical sterilization (bilateral oophorectomy or hysterectomy).

### 4.3.3 Lactation

It is unknown whether Sintilimab and Bevacizumab biosimilar are excreted in human milk. Considering that many drugs are excreted in human milk and the potential for serious adverse reactions in nursing infants from Sintilimab and Bevacizumab biosimilar, women who are breastfeeding are not eligible for this study.

## 4.4 Criteria for Treatment Discontinuation/Withdrawal from Study

### 4.4.1 Discontinuation of Study Treatment

Participants can discontinue treatment at any time for any reason or may be decided by the researcher to discontinue treatment in the event of any adverse event. Furthermore, if a participant is deemed unfit for treatment, violates the study protocol, or for management and/or other safety reasons, the researcher may discontinue the participant's treatment.

Participants must discontinue treatment but may continue to be monitored in the study for any of the following reasons:

- The participant or the participant's legal representative requests discontinuation of treatment.

- An adverse event occurs that requires discontinuation of treatment as specified in the protocol (refer to Section 5.4).

- Another malignancy requiring active treatment occurs.

- A concurrent illness that prevents further treatment occurs.

- The researcher decides to withdraw the participant from the study.

- The participant tests positive in a serum pregnancy test.

- The participant has poor compliance.

- The researcher believes that continuing the study drug would pose an unnecessary risk to the participant based on their health status or personal circumstances.

- Completion of the treatment specified in the protocol.

Visits should be conducted for participants who discontinue treatment but continue to be monitored in the study.

### 4.4.2 Withdrawal from the Study

If the participant or the participant's legal representative withdraws the informed consent, the participant must withdraw from the study. If a participant withdraws from the study, they will no longer receive treatment or attend planned visits. With the participant's consent, they may receive survival follow-up after withdrawal from the study. If a participant is lost to follow-up, they must withdraw from the study.

# 5 Study Treatment

## 5.1 Treatment Plan

Eligible subjects will receive frontline Sintilimab 200 mg IV on day 1 every 3 weeks combined with a biosimilar of Bevacizumab 15 mg/kg IV on day 1 every 3 weeks. Efficacy and surgical resection assessment will be conducted every 6 weeks within the first 6 months after the first medication according to RECIST 1.1 criteria (evaluated by researchers at each center), and thereafter every 9 weeks:

1) If at baseline the patient is CNLC-IIa and technically resectable, after two cycles of drug treatment, if assessed as PR (Partial Response) or SD (Stable Disease) and considered surgically resectable, discontinue the treatment with the biosimilar of Bevacizumab, continue with one cycle of Sintilimab treatment (i.e., at least a 6-week interval between the last dose of biosimilar of Bevacizumab and the surgery), followed by a preoperative assessment and surgical resection. Continue with adjuvant therapy of Sintilimab combined with biosimilar of Bevacizumab for 4-8 weeks post-surgery until recurrence, intolerable toxicity, or up to a maximum of 12 months (16 cycles).

If at baseline the patient is CNLC-IIb, or the tumor is technically unresectable, after drug treatment, if assessed as PR or SD in two consecutive evaluations, and considered surgically resectable, discontinue the treatment with the biosimilar of Bevacizumab, continue with one cycle of Sintilimab treatment (i.e., at least a 6-week interval between the last dose of biosimilar of Bevacizumab and the surgery), followed by a preoperative assessment and surgical resection. Continue with adjuvant therapy of Sintilimab combined with biosimilar of Bevacizumab for 4-8 weeks post-surgery until recurrence, intolerable toxicity, or up to a maximum of 12 months (16 cycles).

2) If PD (Progressive Disease) is assessed during treatment, it is recommended to discontinue the study group and choose another treatment plan;

3) If the assessment result is PR or SD but the surgical evaluation is inoperable, or the assessment result is CR (Complete Response); continue the original treatment plan until toxicity becomes intolerable, disease progression, or death, for a maximum treatment duration of 24 months (32 cycles).

## 5.2 Use of Study Drugs

The study drugs in this research are defined as Sintilimab and a biosimilar of Bevacizumab. The administration plan is as follows (referred to in Table 2).

Table 2: Investigational Therapeutic Drugs and Administration Schemes

| Medication | Dosage/Usage | Dosing Frequency | Administration Method | Treatment Duration/Cycle |
| --- | --- | --- | --- | --- |
| Sintilimab | 200mg | Q3W | Intravenous Infusion | the first day of each 21-day cycle and continued thereafter |
| IBI 310 (Bevacizumab biosimilar) | 15mg/kg | Q3W | Intravenous Infusion | the first day of each 21-day cycle and continued thereafter |

If the biosimilar of Bevacizumab is temporarily discontinued due to related toxicity, the researcher may decide whether to continue using Sintilimab alone. However, if Sintilimab is temporarily discontinued due to related toxicity, both treatments must be interrupted until the toxicity improves to baseline or at least grade 1 according to the NCI CTCAE criteria. After stopping treatment with the biosimilar of Bevacizumab due to surgery, Sintilimab may be used alone until the appropriate time for surgery, but the use of Sintilimab alone before surgery should not exceed 2 cycles for subjects undergoing surgery. When administering the drugs, try to coordinate the administration of Sintilimab and the biosimilar of Bevacizumab on the same day, and adjust the interval with TACE treatment. First, administer Sintilimab, 200 mg, by intravenous infusion over 30-60 minutes. At least 5 minutes later, administer the biosimilar of Bevacizumab, 15 mg/kg, by intravenous infusion. The first intravenous infusion time of the biosimilar of Bevacizumab should last for 90 minutes. If the first infusion is well-tolerated, the time for the second infusion can be reduced to 60 minutes. If the patient also tolerates the 60-minute infusion well, then all subsequent infusions can be completed in 30 minutes. The first administration time of the study drug is recorded as cycle 1, day 1. For all other study treatment cycles, start the administration on day 1 of each cycle after the pre-administration assessment is completed. For administrative reasons, the medication can be administered within 3 days before or after the planned day 1 of each cycle, according to the researcher's judgment.

### 5.2.1 Sintilimab

In this study, Sintilimab produced by Innovent Biologics (Suzhou) Co., Ltd. is used, with a specification of 100mg per vial. The main active ingredient of Sintilimab is a recombinant fully human monoclonal antibody against the programmed death receptor 1 (PD-1), with a concentration of 10 mg/mL. The product appears as a clear, colorless liquid without any foreign particles, flocculation, or precipitation. The excipients include 140 mmol/L mannitol, 25 mmol/L histidine, 20 mmol/L sodium citrate dihydrate, 50 mmol/L sodium chloride, 0.02 mmol/L disodium edetate (disodium EDTA), and 0.2 mg/mL polysorbate 80, with a pH of 6.0.

The smallest packaging unit of Sintilimab is a box, with each box containing one vial of Sintilimab injection liquid packaged in a vial. The packaging box is printed with the drug name, dosage form, specifications, batch number, expiration date, storage conditions, and other information. The vial and the packaging box label have the same information, but the vial label does not include dosage form, precautions, usage, and dosage information. Sintilimab product should be stored in the dark at 2-8°C, and its shelf life is 24 months. If there are quality issues such as cloudiness or precipitation in the injection liquid, it should be immediately sealed and Innovent Biologics should be notified promptly.

The intravenous infusion time of Sintilimab should be within 30 to 60 minutes. It should not be administered by intravenous bolus or a single rapid intravenous injection. The guidance for drug dilution before administration is as follows:

- Solution preparation and infusion

- Do not shake the vial.

- Before use, allow the vial to reach room temperature (25°C or below).

- After removing the vial from the refrigerator, it can be left at room temperature (25°C or below) for up to 24 hours before dilution.

- Before administration, visually inspect the injection for any suspended particles or discoloration. The product is a clear to slightly opalescent, colorless to pale yellow liquid without foreign particles. If visible particles are observed, discard the vial.

- Withdraw 2 vials of the injection liquid (200mg) at once, transfer to an intravenous infusion bag containing 0.9% (9mg/ml) sodium chloride solution, preparing a final concentration range of 1.5~5.0mg/ml. Gently invert the dilution to mix.

- From a microbiological point of view, once diluted, the product must be used immediately and should not be frozen. Stability studies of the product show that it can be stored for 24 hours in the dark at 2-8°C, which includes up to 6 hours at room temperature (20-25°C) with room light (including the administration time). After refrigeration, the vial and/or intravenous infusion bag must be brought to room temperature before use.

- The infusion line used during infusion must be equipped with a sterile, non-pyrogenic, low protein-binding infusion line filter (pore size 0.2μm). The infusion time is within 30 to 60 minutes.

- Do not use the same infusion line for administering other drugs simultaneously.

- This product is for single use only. Any unused drug remaining in the vial must be discarded.

### 5.2.2 IBI310 (Bevacizumab Biosimilar)

In this study, the biosimilar of Bevacizumab produced by Innovent Biologics (Suzhou) Co., Ltd. is used, with a specification of 4 ml:100 mg per vial. The main active ingredient of the biosimilar of Bevacizumab is a recombinant humanized monoclonal antibody against VEGF. The excipients include 1.64 mg/ml sodium acetate, 50 mg/ml sorbitol, 2 mg/ml polysorbate 80, with a pH of 5.2 (adjusted with acetic acid). It appears as a clear, colorless liquid without foreign particles, flocculation, or precipitation.

The storage condition for the biosimilar of Bevacizumab is tentatively set at 2°C to 8°C, protected from light, with a shelf life of 24 months. The expiration date will be further confirmed based on the results of long-term stability tests. After dilution, the biosimilar of Bevacizumab should be used immediately. If it cannot be administered in a timely manner, the prepared solution can be stored refrigerated (2°C to 8°C) for no more than 48 hours or at 30°C, protected from light, for no more than 24 hours. If the injection solution appears cloudy or has precipitate or any other quality issues, it should be immediately sealed and Innovent Biologics should be notified promptly.

The biosimilar of Bevacizumab and Sintilimab can be administered using the same intravenous infusion line. The preparation and infusion process for the biosimilar of Bevacizumab is as follows:

1. Calculate the volume of medication to be administered at 15 mg/kg and record the time when the preparation starts. The calculation formula is as follows:

Administration volume V(ml) = 15mg/kg × body weight (kg) ÷ 25 mg/ml.

2. Withdraw Vml volume of physiological saline from a 250ml 0.9% (weight/volume) sterile saline IV bag and inject Vml of the biosimilar of Bevacizumab solution into the same sterile saline IV bag.

3. Gently invert the infusion bag to mix, ensuring the uniformity of the medication in the bag and avoiding the generation of foam due to violent shaking.

4. Record the start and end times of the medication administration.

5. After administration, use 0.9% physiological saline to rinse the infusion line.

## 5.3 Dose Modification of Study Drugs

### 5.3.1 General Principles

In the event of adverse events during the study, the researcher should first determine the possible causative medication and adjust the medication based on the most severe adverse event that occurred in the previous cycle. Before administering the study medication on day 1 of each cycle, the subject's hematologic and hepatic and renal functions must meet the administration requirements, and all other toxic reactions must be relieved to CTCAE grade 0-1 level or baseline level (excluding hair loss, fatigue, special provisions in the protocol, or other conditions judged by the researcher to be clinically insignificant). If the subject does not meet the medication standards within the planned cycle interval due to an adverse event, the next administration may be postponed. If it is necessary to pause/permanently stop these study medications due to related toxicity, treatment-related AE, or other reasons, other study medications may be used alone if they meet the corresponding medication standards.

### 5.3.2 Dose Modification of Sintilimab

Dose adjustment of Sintilimab is not allowed throughout the study. The principles for pausing and permanently stopping Sintilimab are shown in Table 3.

**Table 3. Treatment adjustment recommendations for Sintilimab**

| **Adverse events** | **Severity level** | **Adjustments** |
| --- | --- | --- |
| Pneumonia | Grade 2 | Treatment withholding until adverse reaction returns to grade 0– 1 |
|  | Grade 3 or 4 or persistent Grade 2 | Termination |
| Diarrhea and  colitis | Grade 2 or 3 | Treatment withholding until adverse reaction returns to grade 0– 1 |
|  | Grade 4 | Termination |
| Hepatitis | Grade 2, AST or ALT within 3–5 × ULN, or TBIL within 1.5–3 × ULN | Treatment withholding until adverse reaction returns to grade 0– 1 |
|  | Grade 3 or 4, AST or ALT > 5× ULN, or TBIL > 3× ULN | Termination * |
| Nephritis | Grade 2 or 3 increased creatinine | Treatment withholding until adverse reaction returns to grade 0– 1 |
|  | Grade 4 increased creatinine | Termination |
| Endocrine  disorders | Symptomatic grade 2 or 3 hypothyroidism, grade 2 or 3 hyperthyroidism, grade 2 or 3 hypophysitis, grade 2 adrenal insufficiency  Grade 3 hyperglycemia or type I diabetes | Treatment withholding until adverse reaction returns to grade 0– 1 |
|  | Grade 4 hypothyroidism  Grade 4 hyperthyroidism  Grade 4 hypophysitis  Grade 3 or 4 adrenal insufficiency  Grade 4 hyperglycemia or type I diabetes | Termination |
| Skin reactions | Grade 3 | Treatment withholding until adverse reaction returns to grade 0– 1 |
|  | Grade 4 Stevens-Johnson syndrome (SJS) or toxic epidermal necrolysis (TEN) | Termination |
| Thrombocytopenia | Grade 3 | Treatment withholding until adverse reaction returns to grade 0– 1 |
|  | Grade 4 | Termination |
| Other  immune-related adverse reactions | Grade 3 or 4 hyperamylasemia or increased lipase Grade 2 or 3 pancreatitis  Grade 2 myocarditis*  Other Grade 2 or 3 immune-related adverse reactions (first occurrence) | Treatment withholding until recovered to grade 0– 1 |
|  | Grade 4 pancreatitis or relapsed pancreatitis of all grades  Grade 3 or 4 myocarditis**  Grade 3 or 4 encephalitis  Grade 4 other immune-related adverse reactions, first occurrence | Termination |

| **Adverse events** | **Severity level** | **Adjustments** |
| --- | --- | --- |
| Relapsed or  persistent adverse reactions | Relapsed grade 3 or 4 (other than endocrine disorders) Grade 2 or 3 adverse reactions that have not recovered to grade 0– 1 within 12 weeks from the last dose (other than endocrine disorders)  Corticosteroid failing to decrease to ≤ 10 mg/day of prednisone or equivalent within 12 weeks after the last dose | Termination |

If treatment-related adverse reactions do not recover to grade 0-1 or baseline level within 6 weeks after the last dose of Sintilimab, Sintilimab should be permanently discontinued. If corticosteroids are used to treat immune-related adverse reactions, the longest pause in Sintilimab treatment due to corticosteroid tapering should not exceed 12 weeks.

### 5.3.3 Management of Sintilimab-Related Infusion Reactions

Sintilimab may cause severe or life-threatening infusion reactions, including severe hypersensitivity or allergic reactions. Signs and symptoms usually appear during or shortly after drug infusion and are usually fully resolved within 24 hours of completing the infusion. The management guidelines for Sintilimab-related infusion reactions are shown in Table 4.

**Table 4. Guidelines for the management of Sintilimab infusion-related reactions**

| **CTCAE grading** | **Treatments** | **Premedications for subsequent infusions** |
| --- | --- | --- |
| Grade 1  Mild reaction; infusion interruption not indicated; intervention not indicated | According to the patient's medical  indications, monitor the vital signs closely  until the patient is stable as determined by the investigator. | N/A |
| Grade 2  Treatment or infusion interruption required, but responds promptly to timely symptomatic treatment (e.g. antihistamines, non-steroidal anti-inflammatory drugs [NSAIDS], anesthetics, and IV fluids);  prophylactic medications indicated for ≤ 24 h | Stop the infusion and monitor symptoms.  Other appropriate treatments include but are  not limited to:  IV fluids  Antihistamines  NSAIDS  Anesthetics  According to the patient's medical  indications, monitor the vital signs closely  until the patient is stable as determined by the investigator.  If symptoms resolve within 1 h after the  interruption of the infusion, then the infusion will be resumed at 50% of the original  infusion rate (e.g. from 100 mL/h to 50  mL/h). Otherwise, interrupt the treatment until symptoms resolve. Pre-medications should be given for subsequent infusions.  If toxicities at grade 2 occur despite adequate pre-medications, the study drugs should be  terminated. | The following pre-medications are recommended within 1.5 h (± 30 min) before IBI308  infusion:  Diphenhydramine 50 mg PO (or equivalent antihistamines).  Acetaminophen 500– 1000 mg  PO (or equivalent  antipyretics). |
| Grade 3 or 4  Grade 3:  Prolonged (i.e. not rapidly  responsive to symptomatic  medication and/or brief interruption of infusion); recurrence of  symptoms following initial  improvement; hospitalization  indicated for other clinical sequelae (e.g. renal impairment, pulmonary infiltration)  Grade 4:  Life threatening; pressors or  ventilatory support indicated | Discontinue infusion.  Other appropriate treatments include but are  not limited to:  Epinephrine**  IV fluids  Antihistamines  NSAIDS  Anesthetics  Oxygen  Pressors  Corticosteroids  According to the patient's medical  indications, monitor the vital signs closely until the patient is stable as determined by the investigator.  Hospitalization maybe indicated.  **Epinephrine should be used immediately for allergic reactions.  The study drugs should be terminated. | Not applicable. |
| Appropriate first-aid equipment should be provided in the ward and physicians should be available at all times during the administration.  For more information, refer to CTCAE V5.0 [(http://ctep.cancer.gov)](http://ctep.cancer.gov/). | | |

### 5.3.4 Other Allowed Dose Modification for Sintilimab

Interruptions in Sintilimab treatment may occur due to internal/surgical events unrelated to the study treatment or for management reasons. Subjects should restart the study treatment within 3 weeks after the planned interruption. The reason for the interruption should be recorded in the subject's study record.

### 5.3.5 IBI310 (Bevacizumab Biosimilar) Dose Modification

Dose adjustment of the biosimilar of Bevacizumab is not allowed during the study. The dose of biosimilar of Bevacizumab is calculated based on the subject's weight at baseline (before day 1 of administration) and remains unchanged throughout the study unless the subject's weight changes by ≥10% compared to baseline.

### 5.3.6 Management of Infusion Reactions Related to the Biosimilar of Bevacizumab

If a subject experiences an infusion reaction while being administered the drug over 60 minutes, the subsequent infusions will be extended to 90 minutes, and this duration will not change in later infusions. Similarly, if an infusion reaction occurs during a 30-minute infusion, the subsequent infusions will be extended to 60 minutes and will remain unchanged thereafter. Specific management for the first and subsequent infusions can be found in Table 5.

Table 5: Initial and Subsequent Infusions of Bevacizumab Biosimilar

| Initial Infusions | Subsequent Infusions |
| --- | --- |
| Prophylactic premedication is not allowed before the infusion of the bevacizumab biosimilar.  Vital signs (pulse rate, respiratory rate, blood pressure, and body temperature) should be recorded within 60 minutes before the infusion.  The infusion of the bevacizumab biosimilar should be completed within 90 (±15) minutes.  Vital signs should be recorded at the end of the infusion and 2 (±1) hours after the infusion.  Patients should be informed about the possibility of delayed post-infusion symptoms and are required to contact their investigator if such symptoms occur. | If the patient has experienced any infusion-related reactions to a previous infusion, prophylactic administration of antihistamines, antipyretics, and/or analgesics may be done during subsequent administrations based on the investigator's judgment.Vital signs should be recorded within 60 minutes before the infusion.  If the patient tolerated the previous infusion without infusion-related reactions, the bevacizumab biosimilar infusion can be completed within 60 (±10) minutes. However, if the patient experienced infusion-related reactions to the previous infusion, the infusion should be completed within 90 (±15) minutes. If a 60-minute infusion is well-tolerated, subsequent infusions of the bevacizumab biosimilar may be completed in over 30 minutes.  -Vital signs should be recorded at the end of the infusion and 2 (±1) hours after the infusion. |

## 5.4 Management Principles for Toxicity of Immune Checkpoint Inhibitors

Adverse events (AEs) related to Sintilimab exposure may have immunological etiologies due to the mechanism of action of Sintilimab, which involves blocking the hyperactivation of autoimmune functions, resulting in immune-related adverse events (irAEs). These irAEs can occur shortly after the first administration or several months after the last dose of Sintilimab and may affect more than one body system simultaneously, such as immune-related pneumonia, diarrhea/colitis, renal insufficiency, rash, hepatitis, endocrinopathies, and peripheral or central neuropathy. Early detection and initiation of treatment are crucial to reducing complications. Based on current clinical trial data, most irAEs are reversible and can be managed by interrupting Sintilimab, administering corticosteroids, and/or other supportive therapies. If a subject experiences the aforementioned AEs, monitor the subject's symptoms and signs, conduct relevant examinations such as bronchoscopy, endoscopy, or skin biopsy, and differentiate the etiology. If no alternative etiology (such as disease progression, concomitant medication, and infection) is identified and corticosteroids and/or other immunosuppressants are required for treatment (excluding endocrine events such as hyperthyroidism/hypothyroidism, hypophysitis, type 1 diabetes, and adrenal insufficiency which may not require immunosuppressive treatment but are still considered related to Sintilimab-induced autoimmune hyperactivation), the AEs should be considered related to Sintilimab-induced immune system hyperactivation and diagnosed as irAEs. Depending on the severity of the irAE, suspend or permanently discontinue Sintilimab treatment and administer corticosteroids.

## 5.5 Concomitant Medications and Adjunctive Therapy

### 5.5.1 Permitted Concomitant Medications/Adjunctive Therapy

- Medications deemed appropriate by the researcher that comply with the protocol specifications (e.g., for the treatment of disease-related symptoms and various AEs related to treatment).

- Subjects requiring long-term medication for underlying conditions such as hypertension, diabetes may continue their medication.

- Local use of corticosteroids is permitted, such as for topical skin use, eye drops, nasal sprays, inhalation, etc.

### 5.5.2 Prohibited Concomitant Medications/Adjunctive Therapy

Subjects are prohibited from receiving the following treatments during this study:

- Biological treatments with anti-tumor effects (except for cytokines used to treat adverse events caused by chemotherapy) and traditional Chinese medicine with anti-tumor effects.

- Medications with immunomodulatory effects, including but not limited to nonspecific immunomodulators (such as thymosin, interferons, interleukins, immunoglobulins, and intravenous immunoglobulin) and traditional Chinese medicine with immunomodulatory effects, etc.

- Chemotherapy drugs.

- Live vaccines within 30 days prior to the first administration of Sintilimab and during the study. Live vaccines include, but are not limited to measles, mumps, rubella, varicella, yellow fever, rabies, BCG, oral typhoid vaccine. Seasonal influenza vaccines administered by injection are allowed; however, intranasal administration of live attenuated influenza vaccines is not allowed.

- Corticosteroids. The use of inhaled corticosteroids as part of fixed therapy for asthma or chronic obstructive pulmonary disease (COPD) is allowed; corticosteroids used for the treatment of immune-related adverse events are permitted; physiological doses of corticosteroids may be used. Note: Preventive use of corticosteroids to avoid allergic reactions (e.g., pretreatment before administration of intravenous contrast agents or chemotherapy) is allowed.

- Long-term use of vitamin K antagonists such as warfarin and small doses of low molecular weight heparins (such as Enoxaparin 40 mg/day) or heparin.

- Long-term use of antiplatelet aggregation drugs, such as aspirin, dipyridamole, or clopidogrel, etc.

Subjects who need to use any of the above treatments for clinical management, as assessed by the researcher, should be excluded from the trial. Participants may receive other medically necessary treatments as deemed appropriate by the investigator.

5.6 Study Drug Management

### 5.6.1 Storage and Management of Study Drugs

Sintilimab and the IBI310 (biosimilar of Bevacizumab) should be refrigerated at 2-8°C, stored in a dry place protected from light, and not frozen. All study drugs, Sintilimab and the biosimilar of Bevacizumab, are transported to each study center via cold chain. Each study center should designate a specific person responsible for the custody and distribution of the study drugs.

The study drugs should be stored in a refrigerator that can only be opened by authorized personnel. Upon receipt of the drugs, the researcher should verify that the transportation temperature is within the specified range, sign for the drugs after confirming the information is correct, and store them at the prescribed temperature. If there is a temperature excursion during transportation or storage at the study center, the drugs should be isolated and transferred to the specified temperature environment as soon as possible, not used for the subjects temporarily, and promptly reported to Innovent Biologics for further instructions.

### 5.6.2 Retrieval and Destruction of Study Drugs

In this study, containers of used study drugs can be destroyed on-site according to the guidelines and operating procedures established by the study center and local institutions. All unused study drugs must be collected and destroyed uniformly after the study is completed/terminated or after the expiration date.

### 5.6.3 Record Keeping of Study Drugs

Designated personnel at the study center must timely record the receipt, distribution, use, inventory, destruction, retrieval, and damage of study drugs according to relevant regulations and guidelines.

# 6 Study Procedures

## 6.1 Study Plan and Schedule

### 6.1.1 Screening Period

During the screening period (Day -28 to -1), the following study procedures must be completed to ensure the subject is eligible for the study:

- Signing the informed consent form

- Verifying inclusion/exclusion criteria

- Recording demographic data, medical history, and history of liver cancer treatment

- Recording previous and concomitant medication use

- Recording vital signs, height, and weight

- Physical examination

- ECOG PS (Eastern Cooperative Oncology Group Performance Status) scoring

- 12-lead electrocardiogram

- Complete blood count/blood chemistry/urinalysis

- Coagulation function test

- Pregnancy test

- Thyroid function

- Cardiac enzyme spectrum

- Virology antibody testing: HIV antibodies, hepatitis B "two pair half" (HBsAg, HBsAb, HBcAb, HBeAg, HBeAb), and HCV antibodies

- HBV-DNA and HCV-RNA (if applicable)

- Adverse event assessment

- Concomitant medication

- Tumor imaging assessment

- Collection of whole blood samples for biomarker analysis

### 6.1.2 Treatment Period Visits

Complete the following physical examinations as well as laboratory and auxiliary tests within 3 days before each drug administration, before surgery, and when clinically necessary:

- Recording vital signs and weight

- ECOG PS scoring

- 12-lead electrocardiogram

- Complete blood count/blood chemistry/urinalysis

- Thyroid function (for the treatment group)

- Cardiac enzyme spectrum

- HBV-DNA and/or HCV-RNA (if applicable)

- Collection of whole blood samples for biomarker analysis (before surgery or when the disease progresses)

During the treatment period, subjects will receive the following treatments:

- Administration of study drugs

- Surgical treatment (if applicable)

The following information will be collected during the treatment period:

- Adverse event assessment

- Recording concomitant medication

- Tumor imaging assessment (efficacy and surgical resection assessment according to RECIST 1.1 criteria every 6 weeks within the first 6 months, then every 9 weeks thereafter.)

### 6.1.3 Safety Visit

A safety visit will be conducted 30 (±7 days) after the last administration of the study drug or before starting a new anti-tumor treatment (whichever comes first), including the following:

- Recording vital signs

- Weight

- Physical examination

- ECOG PS scoring

- 12-lead electrocardiogram

- Complete blood count/blood chemistry/urinalysis

- Coagulation function test

- Thyroid function

- Cardiac enzyme spectrum

- HBV-DNA and/or HCV-RNA (if applicable)

- Adverse event assessment

- Recording subsequent anti-tumor treatment (if applicable)

### 6.1.4 Survival Follow-up

After the safety visit, subjects enter the survival follow-up period, where contact is made with the subject every 90 days (±7 days) (phone visits are acceptable) to obtain as much information as possible about the survival period and any subsequent systemic anti-tumor treatment. For subjects who stopped study drug treatment for reasons other than disease progression, information related to disease progression should also be obtained as much as possible.

### 6.1.5 Post-Treatment Antitumor Therapy

All new antitumor therapies started after the last administration should be collected as much as possible. After starting new anticancer treatment, participants will enter the survival follow-up phase.

6.2 Other Procedures

### 6.2.1 Discontinuation of Treatment/Withdrawal from Study

Participants who stop treatment/withdraw from the study before the completion of the treatment specified in the study protocol are encouraged to continue follow-up and complete all remaining study visits.

When a participant stops treatment/withdraws from the study, all procedures applicable at the end of treatment should be conducted. Follow-up of any adverse events present at the time of stopping treatment/withdrawal from the study should be conducted in accordance with the safety requirements summarized in section 8.4 (Recording of Adverse Events).

If a participant stops treatment/withdraws from the study for reasons other than disease progression, an imaging assessment should be conducted at the time of treatment termination.

Participants who have completed the treatment specified in the study protocol may stop treatment; after stopping treatment, participants should return to the research center for a safety follow-up visit and then enter the survival follow-up phase of this study.

### 6.2.2 Lost to Follow-Up

If a participant fails to return to the clinic for required study visits and/or the research center is unable to contact the participant, the research center must attempt to contact the participant and reschedule the missed visits. In each missed visit, the researcher or designated person must make every effort to re-establish contact with the participant.

# 7 Study Evaluation

## 7.1 Efficacy Assessment

### 7.1.1 Baseline Tumor Imaging Assessment

The first tumor imaging examination during the screening must be performed within 28 days prior to the first administration of the study drug. Imaging examinations conducted as part of routine clinical practice, if of diagnostic quality and performed within 28 days prior to the first administration of the study drug, can be evaluated by the researcher and used for the oncological assessment during the screening period.

The method used for tumor burden assessment at baseline must be consistent with the method used for each follow-up evaluation (CT or MRI). Examine other affected sites based on symptoms and signs indicated by each subject.

### 7.1.2 Tumor Imaging Assessment During the Study

Throughout the study period, imaging assessments should be performed as closely as possible to the planned schedule. Efficacy and surgical resection assessments are conducted every 6 weeks within the first 6 months after the first medication according to RECIST 1.1 criteria (evaluated by researchers at each center), thereafter switching to evaluations every 9 weeks. Researchers may perform unscheduled imaging assessments at any time if the subject shows clinical instability. Tumor assessments continue until the researcher determines the imaging disease progression defined by RECIST 1.1, toxicity intolerance, withdrawal of informed consent, initiation of new anti-tumor treatment, loss to follow-up, or death, whichever occurs first.

For subjects who are evaluated by the researcher based on RECIST 1.1 and show imaging disease progression (PD) for the first time, if the clinical disease is stable, there is no evidence of rapid imaging progression, and the researcher believes that the subject can continue to benefit from the study drug, treatment may continue under the current study protocol. A repeat imaging assessment must be performed at least 4 weeks (±7 days) apart to confirm. If PD is confirmed upon re-evaluation, the subject should discontinue the study protocol treatment; if progression is not confirmed, continue the study protocol treatment and assess according to the planned imaging examination schedule until imaging confirms PD.

If clinical instability occurs during the study, unscheduled imaging assessments may be performed at any time.

Clinical instability is defined as:

- Symptoms and signs of clinical significance suggesting disease progression (including worsening laboratory test values)

- Decline in ECOG PS score

- Rapid disease progression

- Tumor progression at critical anatomical sites requiring other urgent medical interventions (such as spinal cord compression)

For subjects who discontinue treatment for reasons other than imaging PD defined by the researcher, an imaging assessment should be performed at the end of treatment, followed by imaging evaluations according to the scheduled time points in the protocol, until one of the following events occurs: initiation of a new anti-tumor treatment, objective disease progression, subject withdrawal of ICF, loss to follow-up, or death.

### 7.1.3 Post-Treatment and Follow-up Tumor Imaging Checks

For subjects who complete treatment or discontinue treatment for reasons other than PD, a tumor imaging assessment should be performed at the time of treatment completion/discontinuation. Subsequent imaging assessments should continue according to the planned imaging examination schedule until one of the following occurs: initiation of a new anti-tumor treatment, objective disease progression, death, or the end of the study, whichever comes first. Subsequent survival visits occur every 3 months until death, withdrawal of informed consent, or the end of the study.

## 7.2 Safety Assessment

Safety assessments are conducted by researchers at each center, including:

- Incidence, severity, and relationship to study drugs of all AEs, treatment-related AEs (TRAEs), serious AEs (SAEs), and immune-related AEs (irAEs); researchers assess each subject's AEs during the study and follow-up periods according to NCI CTCAE (Version 5.0) for grading and recording. The characteristics of adverse events are determined based on severity, causality, toxicity grading, and measures taken for trial treatment. For detailed instructions on AE assessment and recording, refer to Section 8;

- Surgical safety assessment: Intraoperative bleeding, postoperative liver failure (PHLF) incidence assessed by the International Study Group of Liver Surgery (ISGLS) 2012, and postoperative complications graded using the modified Clavien-Dindo system;

- Number and proportion of subjects whose treatment and surgery are delayed or discontinued due to the above events.

## 7.3 Biomarker Analysis Process

With the permission of the ethics committee, blood biomarkers are collected to predict the value of treatment efficacy and toxicity reactions. Within 14 days before the first medication and before surgery or disease progression, 10 mL of peripheral blood is collected from the subject for the following tests:

1. Epi CGP ctDNA (pan-cancer genomic testing-ctDNA): Detects single nucleotide variations (SNVs), small fragment insertions and deletions (InDels), copy number variations (CNVs), and structural variations (SVs) in tumor-related genes, analyzes
2. T-cell receptor (TCR) diversity sequencing: This involves analyzing the combination of various TCR clones obtained through sequencing, calculating diversity indices such as TCR evenness and convergence, and analyzing their correlation with therapeutic efficacy and immune-related adverse reactions.

# 8. Safety Reports and Adverse Event Management

## 8.1 Definition of Adverse Events

An Adverse Event (AE) is defined as any unfavorable and unintended medical event that occurs in a participant of a clinical trial after the signing of the informed consent form, regardless of its causality with the study drug. An AE includes, but is not limited to, the following situations:

- Aggravation of pre-existing (before entering the clinical trial) medical conditions/diseases (including worsening symptoms, signs, and laboratory test abnormalities);

- New onset of any adverse medical condition (including symptoms, signs, newly diagnosed diseases);

- Abnormal laboratory results with clinical significance.

## 8.2 Definition of Serious Adverse Events

A Serious Adverse Event (SAE) refers to an adverse event that meets at least one of the following criteria:

- Results in death, excluding deaths caused by disease progression of the study indication.

- Life-threatening (the definition of "life-threatening" refers to a situation where the participant is at risk of death at the time of the AE occurrence, not including AEs that could potentially result in death if they worsen).

- Requires hospitalization or prolongs existing hospitalization, excluding the following situations:

- Rehabilitation facilities

- Sanatoriums

- Routine emergency room treatment

- Day surgeries (e.g., outpatient/daytime/non-bedridden surgeries)

- Hospitalization or extended hospital stay itself is not an SAE if it is not related to the worsening of an AE. For example, hospitalization for pre-existing diseases without the occurrence of a new AE or worsening of the pre-existing disease (e.g., for abnormalities in laboratory results that have persisted since before the clinical trial); hospitalization for other reasons (e.g., annual physical examinations); hospitalization as part of the clinical trial protocol (e.g., procedures required by the protocol); elective hospitalization not related to AE worsening (e.g., elective surgery); treatments or surgeries that were scheduled should be documented in the entire trial protocol and/or participant's baseline data; hospitalization solely for the use of blood products.

- Results in persistent or significant disability/incapacity.

- Results in congenital anomalies/birth defects.

- Other significant medical events: defined as events that compromise the participant or require medical intervention to prevent any of the above conditions from occurring.

## 8.3 Evaluation of Adverse Events

Researchers will evaluate all adverse events according to the NCI CTCAE (Version 5.0). Any changes in CTCAE grade of adverse events will be recorded in the adverse event case report form/worksheet.

All adverse events, regardless of their CTCAE grade, must be assessed for their seriousness.

## 8.4 Recording of Adverse Events

Researchers should record AEs or SAEs using medical terminology/concepts. The use of colloquial language and abbreviations should be avoided. All AEs (including SAEs) must be recorded in the Adverse Event section of the Case Report Form (CRF).

### 8.4.1 Collection and Timing of Adverse Events

Researchers learn about adverse events through non-leading questions asked to the participants. All adverse events, including serious adverse events, are collected from the time the informed consent form is signed until 30 days after the last dose of the drug, regardless of whether the events are observed by the researcher or reported spontaneously by the participants.

From 30 days to 90 days after the last dose, researchers must report all SAEs as well as AEs related to the study drug or the study procedures.

After 90 days following the last dose, researchers should report serious adverse events considered related to the study drug or study procedures.

If a new anti-tumor treatment is initiated within 90 days after the last dose, only serious adverse events related to the study drug need to be recorded thereafter.

### 8.4.2 Follow-Up of Adverse Events

Adverse events should be followed up until they return to baseline or to Grade 0-1, or until the researcher deems follow-up unnecessary for reasonable reasons (e.g., cannot recover or has recovered). If an adverse event does not recover, a reasonable explanation should be recorded in the CRF. The recovery status and date of the participant's AE or SAE, whether related to the study drug or not, should be recorded in the CRF and medical records.

### 8.4.3 Content of AE Records

Researchers must fully record any adverse event, including the diagnosis (if no diagnosis is available, then symptoms, signs, including laboratory test abnormalities), start and end dates and times (if applicable), CTCAE severity grade and changes (for Grade 3 or higher events), whether it is a serious adverse event, actions taken with the study drug, treatments given for the AE, and the outcome of the event, as well as the relationship of the adverse event to the study drug.

For serious adverse events, researchers should also provide the date the AE meets the SAE criteria, the date the researcher became aware of the SAE, the basis for the AE being an SAE, hospitalization dates, discharge dates, possible causes of death, date of death, whether an autopsy was performed, causality assessment with the study process, causality assessment with other drugs, and other possible causes leading to the SAE. The researcher should also provide the basis for the relevance judgment and a description of the SAE. The SAE description should include the participant's number, age, gender, height, weight; the indication for and stage of the disease for which the trial drug was being treated, and related general conditions; the clinical course, development, outcome, and result of the SAE; laboratory test results related to the SAE (test time, unit, and normal range must be provided); past medical history related to the SAE, comorbidities, and their occurrence and duration; medication history related to the SAE, concomitant medications, and their start, duration, and dosage; detailed information about the start, duration, and dosage of the trial drug treatment.

Description of AE recording is as follows:

**Diagnoses, Symptoms, and Signs**

If a diagnosis is already established, it should be recorded on the CRF instead of individual symptoms and signs (for example, record liver failure instead of jaundice, elevated transaminases, and flapping tremor). If at the time of reporting it cannot be determined that the symptoms and signs are caused by the diagnosis, they should be recorded as separate AEs/SAEs. If it is determined that the symptoms and signs are caused by the diagnosis, only the diagnosis should be reported separately, with the symptoms and signs included in the diagnosis. AEs should remove the records of symptoms and signs, while SAEs require follow-up update reports.

**Adverse Events Secondary to Other Events**

Typically, adverse events that are secondary to other events (e.g., caused by other events or clinical sequelae) should record the primary event, unless the secondary event is severe or is a serious adverse event (SAE). However, secondary events of significant clinical significance, if occurring at a different time from the primary event, should be recorded as independent adverse events in the CRF. If the relationship between events is unclear, they should be recorded separately in the CRF.

**Persistent or Recurrent Adverse Events**

Persistent adverse events refer to those that have not alleviated and continue to exist between two evaluation points of the subject. Such adverse events should only be recorded once on the CRF. The initial severity of the event should be recorded and updated when the event worsens to record the most severe degree of the event.

Recurrent adverse events refer to those that have alleviated between two evaluation points but occur again later. Each occurrence of the event should be recorded separately in the CRF.

**Laboratory Test Abnormalities**

Clinically significant laboratory test result abnormalities should be reported as AEs. Researchers are responsible for reviewing all abnormal laboratory results and making a medical judgment on whether each laboratory abnormality should be reported as an AE.

**Death**

All deaths occurring during the entire trial period, including the 90-day follow-up period after the last dose, regardless of their relation to the study drug, should be recorded on the CRF's death report form and reported promptly.

When recording a death event, if the cause of death is clear, the cause of death should be recorded as an adverse event, with the outcome of the event being death, and the event reported as an SAE (deaths caused by tumor progression are not recorded and reported as AE/SAE, but researchers should record the death situation on the CRF death report form); if the cause of death is unknown at the time of reporting, it should be recorded on the CRF's adverse event form as "death of unknown cause," and initially reported as an SAE for further investigation of the exact cause of death.

**Pre-existing Medical Conditions**

Symptoms/signs that the subject had during the trial screening period should only be recorded and reported as adverse events if there is an aggravation in severity, frequency, or nature (excluding the worsening of the disease condition being studied). The record should reflect a change relative to the previous state, for example, "increased frequency of headaches."

**Disease Progression**

Disease progression is defined as the worsening of the condition of subjects caused by the primary tumor targeted by the experimental medication, the appearance of new lesions relative to the primary tumor, or the progression of existing lesions. Expected disease progression is not reported as an AE; deaths, life-threatening conditions, hospitalization or prolonged hospitalization, permanent or serious disability/incapacity, congenital anomalies/birth defects, and other significant medical events caused by symptoms and signs of expected disease progression are not reported as SAEs for expedited reporting.

**New Antitumor Treatment**

Within 90 days after the last dose, if the subject begins a new antitumor treatment, only serious adverse events considered to be related to the study drug should be recorded and reported.

## 8.5 Rapid Reporting of SAE and Pregnancy

**SAE Reporting:**

The reporting period for SAEs extends from the signing of informed consent to 30 days (inclusive) after the last dose of medication for serious adverse events that occur. If an SAE occurs, whether it is the initial report or a follow-up report, the investigator must immediately complete the "Serious Adverse Event Report Form" and report it to Innovent within 24 hours of becoming aware at drugsafety@innoventbio.com, and report to the national regulatory authorities and ethics committees as required by Chinese regulations.

Serious adverse events occurring outside the above period that are determined to be related to the study drug should also be reported.

**Pregnancy:**

Given the embryotoxicity safety risks associated with similar drugs, all trial participants of childbearing potential must use effective contraception.

If a female participant becomes pregnant during the clinical trial with drug exposure, the participant will be withdrawn from the study, and the pregnancy must be reported to Innovent within 24 hours of the investigator becoming aware, and the "Innovent Clinical Trial Pregnancy Report/Follow-up Form" must be completed. If the partner of a male participant with drug exposure during the clinical trial becomes pregnant, the participant may continue in the clinical trial, and the pregnancy must be reported to Innovent within 24 hours of the investigator becoming aware, and the "Innovent Clinical Trial Pregnancy Report/Follow-up Form" must be completed.

Investigators must continuously monitor and follow up on the pregnancy outcomes of participants who become pregnant, following up until 8 weeks after childbirth, and report the outcomes to Innovent.

If the pregnancy results in stillbirth, spontaneous abortion, fetal malformation (any congenital anomalies/birth defects), or medically induced abortion for medical reasons, it is considered an SAE and must be reported according to SAE reporting procedures and timelines.

If an SAE occurs during pregnancy, it must be reported following the SAE reporting procedures.

## 8.6 Compensation for Injury

The sponsor has purchased relevant insurance for all participants in this study. If a life-threatening adverse event occurs during the study, with reasonable evidence suggesting a direct causal relationship with the provided experimental drug and is an unexpected adverse reaction, the investigator will provide reasonable compensation for the injury according to legal regulations. The above compensation costs will not be reimbursed by the study if covered by the participant's medical insurance.

# 9 Statistical Analysis

## 9.1 Statistical Hypotheses and Sample Size

This is an exploratory Phase II study, not involving hypothesis testing or sample size estimation, with an expected total enrollment of 30 participants.

## 9.2 Statistical Analysis Methods

### 9.2.1 General Statistical Analysis Methods

All statistical analyses will be performed using SAS 9.2 (or higher version) statistical analysis software. All statistical tests will be one-sided with an alpha level of 0.05 for superiority tests, and comparisons between groups will provide 95% confidence intervals and p-values.

Unless otherwise specified, quantitative data will be described using mean ± standard deviation or median (minimum, maximum). Count data will be described using frequencies (percentages); Kaplan-Meier estimates will be used for mEFS, mRFS, mPFS, and mOS.

### 9.2.2 Efficacy Analysis

- Event-Free Survival (EFS) is defined as the time from enrollment to disease progression, recurrence, or death (whichever occurs first);

- Conversion resection rate: defined as the proportion of participants undergoing curative resection out of the total participants;

- Pathological remission rate: the proportion of tumor necrosis shown in surgical resection specimens, judged by pathology as the standard;

- Objective Response Rate (ORR) is defined as the proportion of participants with CR and PR out of the total participants;

- Recurrence-Free Survival (RFS) is defined as the time from curative resection to tumor recurrence or death (whichever occurs first);

- Progression-Free Survival (PFS) is defined as the time from enrollment to disease progression or death (whichever occurs first).

- Overall Survival (OS) is defined as the time from enrollment to death from any cause. EFS, ORR, RFS, and PFS will be assessed by the investigators according to RECIST 1.1 standards.

### 9.2.3 Safety Analysis

During the study and follow-up period, the safety of drug treatment and surgical procedures will be evaluated. This includes the incidence, severity, and the relationship with the study drug, as well as the number and proportion of participants whose treatment and surgery were delayed or discontinued due to the aforementioned events.

### 9.2.4 Exploratory Analysis

Blood biomarkers (including pan-cancer genomic testing - ctDNA and T cell receptor diversity sequencing) will be used to predict the value of treatment efficacy and adverse reactions before and after medication.

# 10 Quality Assurance and Quality Control

Researchers should implement and maintain a quality assurance and quality control system according to the relevant standard operating procedures to ensure that the implementation of clinical trials and the collection, recording, and reporting of data comply with the protocol, Good Clinical Practice (GCP), and relevant regulatory requirements.

# 11 Ethics

## 11.1 Ethics Committee

Researchers are responsible for preparing and submitting relevant documents to the ethics committee (EC) of the research center, including the trial protocol, informed consent form (ICF), investigator's brochure, subject recruitment materials or advertisements, and other regulatory-required documents for EC approval. Written approval from the EC must be obtained before starting the study. The EC's approval document must specify the name, number, version number of the study protocol, and the version number of other documents (e.g., informed consent form) and the approval date.

## 11.2 Ethics of This Study

The process of the study and obtaining informed consent should comply with the Declaration of Helsinki, relevant GCP requirements, and China's laws and regulations on drugs and data protection.

GCP provides ethical and scientific global quality standards for the design, implementation, recording, and reporting of clinical research involving human subjects. This study will be conducted in accordance with GCP and relevant national regulations, and comply with the ethical principles in the Declaration of Helsinki to protect the rights, safety, and health of subjects.

Researchers must follow the processes stipulated in the trial protocol, and any violations will be reported to the EC or regulatory authorities.

## 11.3 Subject Information and Informed Consent

Before any study procedure begins, potential subjects will be explained the possible risks and benefits of the study using the ICF in simple and understandable language. The ICF declaration should specify that informed consent is voluntarily signed, and clarify the possible risks and benefits of participating in the study, and that subjects can withdraw from the study at any time. Researchers can only enroll subjects after thoroughly explaining the detailed content of the study, satisfactorily answering the subjects' questions, and giving them enough time to consider, and obtaining written consent from the subjects or their legal representatives.

Researchers are responsible for explaining the content of informed consent to subjects and obtaining the signed and dated informed consent form from the subjects or their legal representatives before the study begins. After signing, researchers should provide subjects with a copy of the signed informed consent form.

The informed consent process should be documented in the trial master file.

## 11.4 Protection of Subject Data

The ICF will include (or in some cases, along with a separate document) information related to data protection and privacy. Precautions will be taken to ensure the confidentiality of documents and prevent identification of subjects. However, in special circumstances, such as medical emergencies, some individuals may see a subject's genetic data and personal identification codes. In addition, relevant regulatory authorities may require access to related documents.

# 12 Study Management

## 12.1 Data Handling and Record Retention

Documents in the clinical trial (protocol and protocol amendments, completed CRFs, signed ICFs, etc.) should be retained and managed according to GCP requirements.

Research documents should be reasonably preserved for future access or data traceability, considering safety and environmental risks.

## 12.2 Access to Source Data/Documents

Researchers agree to allow direct access to all study-related documents, including subject medical records, by relevant regulatory authorities.

## 12.3 Protocol Amendments

All amendments to the protocol should be kept as protocol supplements. Any modification to the protocol must be submitted to the ethics committee for approval or filing according to the regulations of the ethics committee.

## 12.4 Responsibilities of the Investigator

The investigator will conduct this study following the protocol, the ethical principles of the Declaration of Helsinki, Chinese GCP, and relevant regulatory requirements.
