# Supplementary material for: Sintilimab plus bevacizumab followed by resection in intermediate-stage hepatocellular carcinoma: a phase Ib clinical trial with biomarker analysis
Source: BMJ Oncol. 2024 Dec 16;3(1):e000578. doi: 10.1136/bmjonc-2024-000578 (PMC11880783; doi:10.1136/bmjonc-2024-000578)
Supplement: online supplemental file 2 [file bmjonc-3-1-s002.pdf]

Supplementary materials

Content

Inclusion Criteria 2
Exclusion Criteria 2
Supplement methods........ 5
Supplementary Table 1. Best overall response assessed by investigators........ 8
Supplementary Table 2. Treatment-related adverse events of any grade and of grade 3....... 9
Supplementary Figure 1. Predictive role of CNV score in patients' survival outcome and tumor response........ 10
Supplementary Figure 2. Predictive role of tumor fraction in patients' survival outcome and tumor response. 11
Supplementary Figure 3. Predictive role of CNV score and tumor fraction in tumor recurrence after hepatectomy........ 12
Supplementary Figure 4. Predictive role of TCR clone number in patients' survival outcome. 13

Inclusion Criteria

1. Able to provide informed consent and willing to sign an approved consent form before any trial-related procedures are conducted.
2. Male or female, aged ⩾18 and ⩽75 years.
3. Hepatocellular carcinoma diagnosed with histology/cytology or clinically according to Guidelines for the Diagnosis and Treatment of Hepatocellular Carcinoma (2019 Edition).
4. Intermediate-stage HCC (BCLC stage B or CNLC stage IIa/IIb) deemed potentially resectable upon the investigators' assessment.
5. No prior treatment for HCC.
6. Child-Pugh class A.
7. Eastern Cooperative Oncology Group Performance Status (ECOG PS) score of 0-1.
8. Expected survival time of more than 6 months.
9. At least 1 measurable lesion according to RECIST 1.1 criteria.
10. Adequate bone marrow and organ function, including:

    1) Absolute Neutrophil Count (ANC) $\geqslant 1.0\times10^9$/L without the use of granulocyte colony-stimulating factor within the last 14 days;

    2) Platelets $\geqslant 75\times10^9$/L without transfusion within the last 14 days;

    3) Hemoglobin ⩾9 g/dL without transfusion or the use of erythropoiesis-stimulating agents within the last 14 days;

    4) Total bilirubin ⩽2.0 times the upper limit of normal (ULN);

    5) Albumin ⩾2.8 g/dL;

    6) Aspartate aminotransferase (AST) and Alanine aminotransferase (ALT) within ⩽5 times ULN;

    7) Creatinine ⩽1.5 times ULN and creatinine clearance (calculated using the Cockcroft-Gault formula) ⩾30 mL/min;

    8) International Normalized Ratio (INR) or Prothrombin Time (PT) ⩽1.5 times ULN.

11. For female participants of childbearing potential, must undergo a blood pregnancy test within the first 3 days of randomization with negative results and agree to use a reliable and effective method of contraception during the trial and within 120 days of the last trial drug administration. Male patients whose partners are women of childbearing age must agree to use a reliable and effective method of contraception during the trial and within 120 days of the last trial drug administration.
12. All participants (both male and female) who are at risk of conceiving must use a contraceptive method with a failure rate of less than 1% per year throughout the treatment period and for 120 days after the last administration of the study drug (or 180 days after the last administration of study drug).

Exclusion Criteria

1. Cholangiocarcinoma (ICC), sarcomatoid hepatocellular carcinoma, and hepatic fibrolamellar carcinoma.
2. History of organ transplantation or hepatic encephalopathy.
3. Tumor burden exceeding 70% of liver volume.
4. Presence of clinically symptomatic pleural effusion, ascites, or pericardial effusion requiring drainage.

5. History of any renal disease or nephrotic syndrome.
6. History of esophageal or gastric variceal bleeding due to portal hypertension in the past 6 months; known severe (G3) varices from endoscopy within 3 months before first administration; evidence of portal hypertension (including imaging findings of splenomegaly with a longitudinal diameter over 10 cm and platelets below 100×10^9/L), with high bleeding risk assessed by the investigators.
7. Any life-threatening bleeding event in the past 3 months, including those requiring transfusion treatment, surgery, or local therapy, ongoing medication treatment.
8. History of arterial or venous thromboembolic events in the past 6 months, including myocardial infarction, unstable angina, cerebrovascular accident or transient ischemic attack, pulmonary embolism, deep vein thrombosis, or any other serious thromboembolic disease. Exceptions include catheter-related thrombosis or superficial vein thrombosis that has stabilized after conventional anticoagulant therapy.
9. Severe bleeding tendency or coagulopathy, or are receiving thrombolytic therapy.
10. Need for long-term use of vitamin K antagonists (such as warfarin) or low-dose low molecular weight heparin (such as enoxaparin 40 mg/day) or heparin.
11. Need for long-term use of drugs that can inhibit platelet function, such as aspirin, dipyridamole, or clopidogrel.
12. Uncontrollable hypertension, with systolic blood pressure >140 mmHg or diastolic blood pressure >90 mmHg after optimal medical treatment, history of hypertensive crisis, or hypertensive encephalopathy.
13. Symptomatic congestive heart failure (New York Heart Association Class II-IV), symptomatic or poorly controlled arrhythmia, history of congenital long QT syndrome or screening corrected QTc >500 ms (calculated using Fridericia's formula).
14. History of gastrointestinal perforation and/or fistula, intestinal obstruction (including partial intestinal obstruction requiring parenteral nutrition), extensive bowel resection (partial colectomy or extensive small bowel resection with chronic diarrhea), Crohn's disease, ulcerative colitis, or long-term chronic diarrhea within the past 6 months.
15. Major surgical procedures (craniotomy, thoracotomy, or laparotomy) or unhealed wounds, ulcers, or fractures within 4 weeks prior to the first administration; tissue biopsy or other minor surgeries within 7 days prior to the first administration, except for venous catheterization for intravenous infusion.
16. History of pulmonary fibrosis, interstitial pneumonia, pneumoconiosis, drug-related pneumonia, or other severe lung diseases with significant impairment of lung function.
17. Active acute or chronic hepatitis B or C infection. Hepatitis C virus (HCV) RNA $>10^3$ copies/mL; positive for both hepatitis B surface antigen (HbsAg) and anti-HCV antibodies; hepatitis B virus (HBV) DNA positive but has received antiviral treatment can be enrolled
18. Active tuberculosis, undergoing anti-tuberculosis treatment or having received anti-tuberculosis treatment within 1 year prior to the first administration.
19. Human immunodeficiency virus (HIV) infection (positive for HIV 1/2 antibodies), known syphilis infection.
20. Severe infection in active phase or poorly controlled clinically. Severe infection within 4 weeks prior to the first administration, including but not limited to hospitalization for complications due to infection, bacteremia, or severe pneumonia.

21. Active autoimmune disease requiring systemic treatment (e.g., disease-modifying drugs, corticosteroids, or immunosuppressants) within 2 years prior to the first administration; replacement therapy allowed (e.g., thyroxine, insulin, or physiological corticosteroids for adrenal or pituitary insufficiency, etc.); known primary immunodeficiency; subjects with only positive autoimmune antibodies need to be assessed by the researcher to confirm the presence of autoimmune disease.
22. Use of immunosuppressive drugs within 4 weeks prior to the first administration, excluding nasal, inhaled, or other local routes of corticosteroids or physiological doses of systemic corticosteroids (i.e., no more than 10 mg/day of prednisone or an equivalent dose of other corticosteroids), allowed for temporary use for symptoms of respiratory distress due to diseases such as asthma, chronic obstructive pulmonary disease, etc.
23. Received live attenuated vaccines within 4 weeks before the first dose or plan to receive live attenuated vaccines during the study period.
24. Received traditional Chinese medicine with anti-tumor indications or drugs with immunomodulatory effects (including thymosin, interferon, interleukin, except for local use to control pleural effusion or ascites) within 2 weeks before the first administration.
25. Uncontrolled/correctable metabolic disorders or other non-malignant organ diseases or systemic diseases or cancer-related reactions, which could lead to higher medical risks and/or uncertainty in survival evaluation.
26. Diagnosed with other malignancies within 5 years before the first administration, excluding adequately treated basal cell carcinoma of the skin, squamous cell carcinoma of the skin, and/or in situ carcinoma that has been radically resected. If diagnosed with other malignancies more than 5 years before administration, pathological or cytological diagnosis of recurrent or metastatic lesions is required.
27. Previous treatment with any anti-PD-1, anti-PD-L1/L2, anti-CTLA4 antibodies, or other immunotherapy.
28. Known allergy to sintilimab or bevacizumab or any excipients, or severe allergic reactions to other monoclonal antibodies.
29. Participation in other clinical trial treatments within 4 weeks before the first administration.
30. Pregnant or breastfeeding female patients.
31. Other acute or chronic diseases, mental illnesses, or abnormal laboratory test values that could result in increased related risks of participating in the study or administering the study drug, or interfere with the interpretation of the study results, and the patient is deemed ineligible to participate in the study by the researcher.

Supplement methods

Blood sample collection, cell-free DNA (cfDNA) and genomic DNA isolation

Blood samples were collected at baseline (1–3 days before sintilimab/bev treatment initiation), post-systemic therapy (1–3 days after disease progression or 1-3 days before hepatectomy) and post-surgery (4-6 weeks after surgery), in two 10-mL Cell-Free DNA BCT tubes (Streck). Plasma and buffy coat were separated within 1–4 days via two centrifugation at room temperature for 10 minutes (1600 × g and 16000 × g, respectively) and then stored at -80C°.

Both cfDNA and genomic DNA samples were quantitated by the Qubit 1x dsDNA High Sensitivity Assay (Thermo Fisher Scientific). A predefined minimum yield of 100 ng of genomic DNA or 20 ng of cfDNA was used as acceptance criteria for the downstream library preparation. cfDNA samples were further subject to quality control using Agilent 4200 Tapestation and cell-free DNA ScreenTape assay (Agilent). Samples with %cfDNA greater than 70% were included for downstream processes to ensure successful library preparation and sequencing.

ctDNA low-pass whole genome sequencing, targeted sequencing and bioinformatic analyses

ctDNA sequencing was performed at Epione Medlab, a CAP-accredited laboratory. 20-30ng of cfDNA was used to construct whole genome sequencing (WGS) libraries using KAPA HyperPrep Kit (Roche). An aliquot of the libraries was directly sequenced to a mean depth of ~2x on NovaSeq 6000 (Illumina) (PE150) for copy number variation (CNV) and fragment analyses. Samples with Q30 above 85% and mapping rate above 90% were used for further analyses. Another aliquot of the libraries was hybridized to custom-designed target enrichment probes (EpiCGP 500) following KAPA HyperCap Workflow v3.0 (Roche). The final libraries were sequenced on NovaSeq 6000 (Illumina) to the range of raw depths between 25,870x to 90,847x for somatic mutation detection and analyses.

For the analyses of low-pass WGS sequencing data, Trim Galore (https://www.bioinformatics.babraham.ac.uk/projects/trim_galore/) was used for adapter removal, filtering of low-quality reads, and trimming, to generate clean reads. The clean reads were then aligned to the human genome reference sequence hg19 with BWA-MEM.1 After that, the data were processed with GATK2 to obtain deduplicated data, which were then used as input for IchorCNA3 to detect genomic alterations such as CNV and tumor fraction. To ensure high data quality, only samples with a GC-Map correction MAD (median absolute deviation) below 0.15 and mean coverage above 0.1x were included. Finally, a custom pipeline was developed to determine the CNV Score by the Shannon's entropy formula, which represents the overall fluctuation or disorder of the CNV on a genome-wide scale. There have been studies using the CNV Score to characterize the extent of genomic aberrations in tumor patients.4, 5 The tumor fractions indicating the amount of tumor-derived DNA fragments in plasma cfDNA3 were obtained from the analysis results of IchorCNA software. The molecular tumor burden (MTB) is a probability value predicted by a machine learning model based on tumor fraction, CNV Score and the ctDNA fragmentation distribution.

For the analyses of EpiCGP 500 targeted sequencing data, variants were called using Vardict v1.66 and Varscan v2.4.2,7 incorporating bioinformatic methods from FGBIO v0.9.0 (http://fulcrumgenomics.github.io/fgbio/) and iDES (integrated digital error suppression)8 to remove PCR duplicates and stereotypical errors. Somatic variants were derived after filtering out

common germline variants in public databases, including ExAC, dbSNP, and 1000 Genomes. Only nonsynonymous SNVs or INDELs with an allele frequency (AF) of at least 0.3% were included in further analyses. The ctDNA mutations were then identified after removing spontaneous mutations based on a custom database. Maximum somatic variant allelic frequency (maxVAF) was defined as the maximum VAF of all ctDNA mutations identified in each cfDNA sample.9

TCR Repertoire Analyses

200 ng of genomic DNA was amplified using the Oncomine TCR Beta-SR Assay (Thermo Fisher Scientific). Products were purified with Agencourt AMPure XP beads (Beckman Coulter), washed with 70% ethanol, and eluted in 30 µL Low TE buffer. Resulting libraries were quantified using the Ion Library Quantitation Kit (Thermo Fisher Scientific), then diluted to 40 pM. Equal volumes from up to 32 samples were pooled together for sequencing on one Ion 540 chip.

The Ion Torrent S5XL generated raw sequence data in the bcl format, which were demultiplexed and preprocessed utilizing the BaseCaller plugin to generate sample-specific UBAM files. Data were further analyzed using the Ion Reporter™ Software v5.14, including filtering of low-quality and off-target reads, removal or correction of error-containing reads, annotation of rearrangements by alignment to IMGT database, which is a pioneering international information system in immunogenetics and immunoinformatics (https://www.imgt.org/), identification and elimination of indel and PCR errors, and reporting of VDJ rearrangements and secondary analysis of repertoire features.

References

1. Li H, Durbin R. Fast and accurate short read alignment with Burrows-Wheeler transform. Bioinformatics. 2009;25(14):1754-1760. doi:10.1093/bioinformatics/btp324
2. McKenna A, Hanna M, Banks E, et al. The Genome Analysis Toolkit: a MapReduce framework for analyzing next-generation DNA sequencing data. Genome Res. 2010;20(9):1297-1303. doi:10.1101/gr.107524.110
3. Adalsteinsson VA, Ha G, Freeman SS, et al. Scalable whole-exome sequencing of cell-free DNA reveals high concordance with metastatic tumors. Nat Commun. 2017;8(1):1324. doi:10.1038/s41467-017-00965-y
4. Freire P, Vilela M, Deus H, et al. Exploratory analysis of the copy number alterations in glioblastoma multiforme. PloS One. 2008;3(12):e4076. doi:10.1371/journal.pone.0004076
5. Obulkasim A, Ylstra B, van Essen HF, et al. Reduced genomic tumor heterogeneity after neoadjuvant chemotherapy is related to favorable outcome in patients with esophageal adenocarcinoma. Oncotarget. 2016;7(28):44084-44095. doi:10.18632/oncotarget.9857
6. Lai Z, Markovets A, Ahdesmaki M, et al. VarDict: a novel and versatile variant caller for next-generation sequencing in cancer research. Nucleic Acids Res. Jun 20 2016;44(11):e108. doi:10.1093/nar/gkw227
7. Koboldt DC, Zhang Q, Larson DE, et al. VarScan 2: somatic mutation and copy number alteration discovery in cancer by exome sequencing. Genome Res. Mar 2012;22(3):568-76. doi:10.1101/gr.129684.111
8. Newman AM, Lovejoy AF, Klass DM, et al. Integrated digital error suppression for improved detection of circulating tumor DNA. Nat Biotechnol. May 2016;34(5):547-555. doi:10.1038/nbt.3520

9. Li H, Di Y, Li J, et al. Blood-based Genomic Profiling of Circulating Tumor DNA from Patients with Advanced Pancreatic Cancer and its Value to Guide Clinical Treatment. Research Paper. Journal of Cancer. 2020;11(15):4316-4323. doi:10.7150/jca.43087

Supplementary Table 1. Best overall response assessed by investigators.

	RECIST v1.1<br>N=30	mRECIST<br>N=30
**CR (%)**	0 (0.0%)	2 (6.7%)
**PR (%)**	8 (26.7%)	9 (30.0%)
**SD (%)**	19 (63.3%)	16 (53.3%)
**PD (%)**	3 (10.0%)	3 (10.0%)
**ORR (%)**	26.7%	36.7%

Supplementary Table 2. Treatment-related adverse events of any grade and of grade 3.

	**Any grade**	**Grade 3**
All adverse events	25 (83.3%)	7 (23.3%)
Hypertension	13 (43.3%)	2 (6.7%)
Proteinuria	11 (36.7%)	2 (6.7%)
Aspartate aminotransferase increased	6 (20.0%)	1 (3.3%)
Periodontal disease	6 (20.0%)	0
Dermatitis	6 (20.0%)	1 (3.3%)
Alanine aminotransferase increased	4 (13.3%)	1 (3.3%)
Adrenal insufficiency	3 (10.0%)	0
Blood bilirubin increased	2 (6.7%)	0
Fatigue	2 (6.7%)	0
Anorexia	2 (6.7%)	0
Pyrexia	1 (3.3%)	0
Arthritis	1 (3.3%)	0
Hypothyroidism	1 (3.3%)	0
Atrial fibrillation	1 (3.3%)	0
Platelet count decreased	1 (3.3%)	0

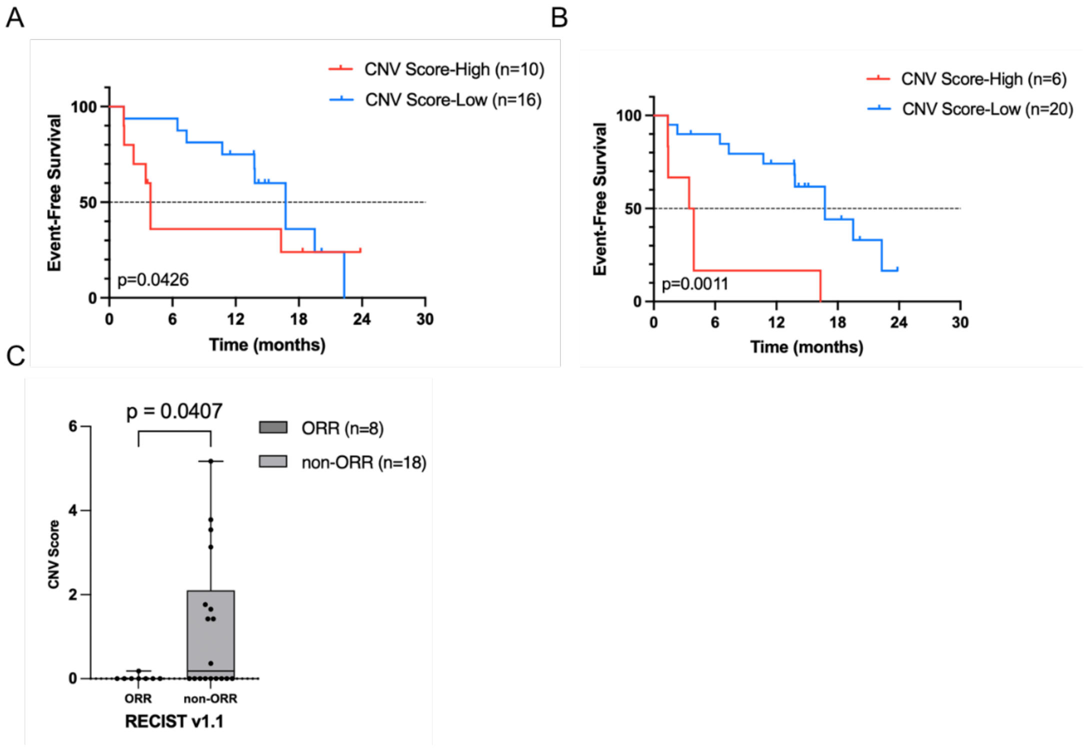

Supplementary Figure 1. Predictive role of CNV score in patients' survival outcome and tumor response.

(A) Kaplan-Meier survival analysis shows the probability of EFS stratified by CNV score of baseline. (B) Kaplan-Meier survival analysis shows the probability of EFS stratified by CNV score of post-systemic therapy samples. (C) CNV score in patients who did or did not achieve objective response.

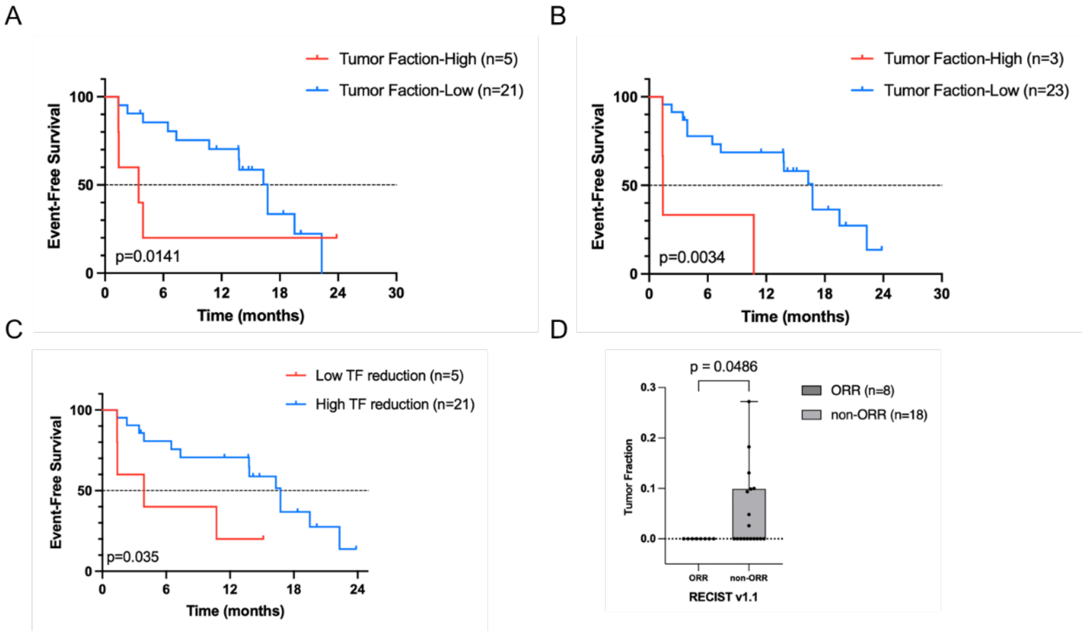

Supplementary Figure 2. Predictive role of tumor fraction in patients' survival outcome and tumor response. (A) Kaplan-Meier survival analysis shows the probability of EFS stratified by tumor fraction of baseline. (B) Kaplan-Meier survival analysis shows the probability of EFS stratified by tumor fraction of post-systemic therapy samples. (C) Kaplan-Meier survival analysis shows the probability of EFS stratified by tumor fraction reduction between baseline and post-systemic therapy. (D) tumor fraction in patients who did or did not achieve objective response.

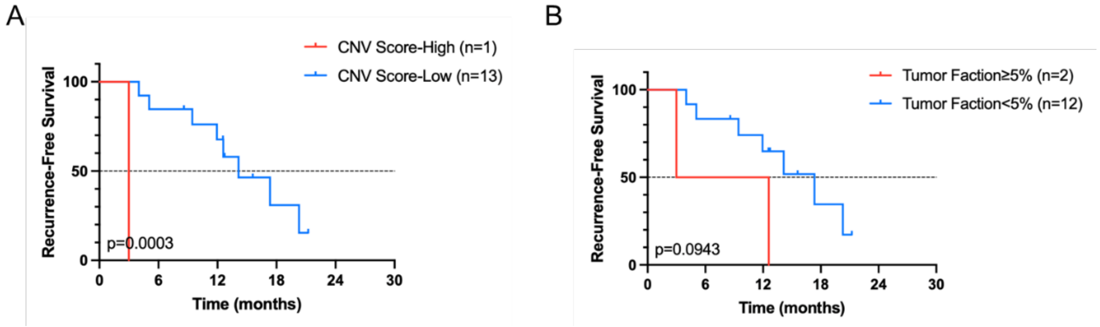

Supplementary Figure 3. Predictive role of CNV score and tumor fraction in tumor recurrence after hepatectomy.

(A) Kaplan-Meier survival analysis shows the probability of RFS stratified by CNV score of post-surgery samples. (B) Kaplan-Meier survival analysis shows the probability of RFS stratified by tumor fraction of post-surgery samples.

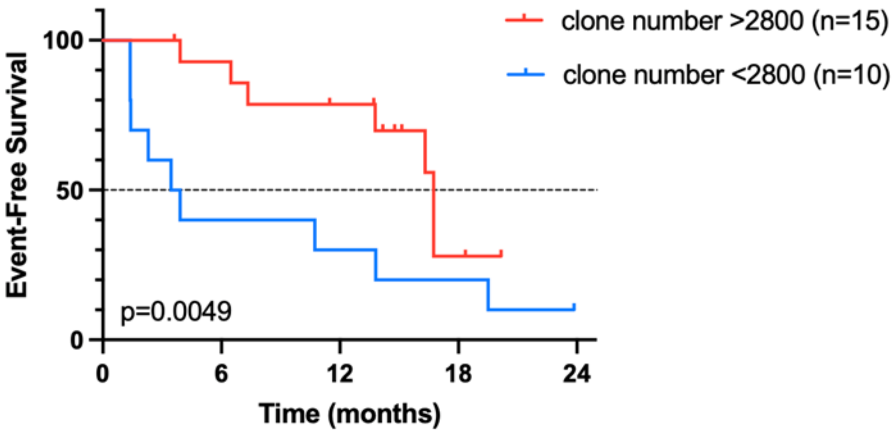

Supplementary Figure 4. Predictive role of TCR clone number in patients' survival outcome.

Kaplan-Meier survival analysis shows the probability of EFS stratified by TCR clone number of post-systemic therapy samples.
